# Optimising electrical vestibular stimulation (EVS) for assessing vestibular function

**DOI:** 10.1016/j.cnp.2025.08.006

**Published:** 2025-09-05

**Authors:** Raphael Hamel, Peter Gaskell, Sophie Prosser, Darren Whelan, Richard Irving, Raymond Francis Reynolds

**Affiliations:** a School of Sport, Exercise, and Rehabilitation Sciences, University of Birmingham, UK; b Interacoustics, Audiometer Alle 1, 5500 Middelfart, Denmark; c University Hospital Birmingham, Centre for Rare Diseases, UK

**Keywords:** Electrical Vestibular Stimulation, Torsional Vestibulo-Ocular Reflex (VOR), Interaural Vestibular Asymmetry, Electrode Montage, Stimulus Frequency

## Abstract

**Objective:**

Electrical vestibular stimulation (EVS) exhibits similar diagnostic performance to caloric irrigation for assessing interaural vestibular asymmetry. To optimise EVS for clinical uses, we investigated the influence of EVS stimulus parameters upon absolute torsional eye response amplitude and asymmetry assessments.

**Methods:**

In young healthy adults (n = 72), monoaural sinusoidal EVS was delivered to each mastoid process for 40 s whilst ocular torsion was assessed. Ambient lights, electrode montage, and stimulus frequency were systematically manipulated.

**Results:**

Ambient lights (250 lx) attenuated torsional responses by 20 % compared to darkness. When the reference electrode was next to C7 (i.e., mastoid-C7 montage), responses to monoaural stimuli were 50 % those of binaural stimulation. Sinusoidal stimuli at 0.5 Hz evoked torsional responses 250 % larger than at 2 Hz but required 300 % more time to estimate asymmetry (12sec vs 4sec). Perceived discomfort was lower at 0.5 Hz than at 2 Hz. Pooled results (n = 53) suggest a normative asymmetry range of 0 ± 25 % for assessing pathological asymmetry.

**Conclusions:**

Darkness and lower stimulus frequencies optimise absolute torsional eye response amplitude. Higher stimulus frequencies accelerate asymmetry assessments. To activate a single vestibular end organ, a mastoid-C7 (or mastoid-acromioclavicular) montage should be used.

**Significance:**

These results extend the clinical potential of EVS for assessing vestibular function.

## Highlights

• We investigated the influence of stimulus parameters upon eye movements evoked by Electrical Vestibular Stimulation.
• Evoked ocular torsion responses were facilitated by darkness, lower stimulus frequency, and appropriate electrode placement.
• These results report optimal stimulation parameters for assessing vestibular function and asymmetry using EVS.

## 1 Introduction

Electrical vestibular stimulation (EVS) non-invasively activates the vestibular system by delivering electrical current to the mastoid processes (Fitzpatrick and Day, 2004), modulating the firing rate of both otolith and semicircular canal afferents (Goldberg et al., 1984). Despite this broad activation, the predominant sensation produced by EVS is one of head rotation around a naso-occipital axis – consistent with selective activation of canal afferents (Day et al., 2011, Day and Fitzpatrick, 2005, Khosravi-Hashemi et al., 2019, Mian et al., 2010, Reynolds and Osler, 2012). Whilst canal afferent activation can evoke behavioural responses such as sway, locomotor adjustments, and orientation responses (Bent et al., 2002, Day et al., 1997, Fitzpatrick et al., 2006, Reynolds, 2011), this study focuses on its oculomotor effects. Specifically, EVS also activates the vestibulo-ocular reflex, predominantly eliciting torsional eye responses with a small translational component (Mackenzie and Reynolds, 2018, Schneider et al., 2002, Schneider et al., 2000, Severac et al., 2003). Translating these oculomotor responses into clinical tools has historically been challenging. However, emerging technologies and protocols now offer a path toward clinical implementation.

Until recently, measuring EVS-evoked ocular torsion required invasive methods such as scleral coils (Aw et al., 2013a, Aw et al., 2008, Aw et al., 2013b) or manually marking the sclera with a pen (Jahn et al., 2003, Schneider et al., 2002), rendering its use as a real-world clinical tool impractical. Advances in eye-tracking technology now enable non-invasive measurement of ocular torsion by tracking iris features using cameras (Jin et al., 2020, Ong and Haslwanter, 2010, Otero-Millan et al., 2015). In recent years, such technology has become commercially available, raising the possibility of implementing EVS to assess vestibular function in clinical settings. In direct support, recent work showed that EVS performs as well as caloric irrigation in estimating interaural asymmetry in patients with unilateral vestibular schwannoma (Mackenzie et al., 2019). However, despite its clinical promise, the optimal EVS parameters for assessing ocular torsion and vestibular asymmetry remain undefined. The overarching aim of this study was to address this gap to further the clinical potential of EVS for assessing vestibular function.

One parameter that could influence EVS-evoked ocular torsion is visual fixation of a target. Target fixation could potentially suppress horizontal and vertical nystagmus (Hirvonen et al., 2012, Korda et al., 2021), facilitating ocular torsion tracking by ensuring the iris image remains stable and within view of the camera. However, it remains unclear whether such fixation is best performed in darkness or with ambient lights. On the one hand, work in rhesus monkeys reported that post-rotational torsional nystagmus is suppressed by 20 % in ambient lights compared to darkness (Straumann et al., 1992), raising the possibility that ambient lights could also reduce EVS-evoked ocular torsion. On the other hand, ambient lights increase iris surface area, providing more features to track and potentially improving torsion tracking accuracy (Jin et al., 2020, Ong and Haslwanter, 2010). Overall, it remains uncertain whether fixation in darkness or with ambient lights provides optimal conditions for tracking EVS-evoked ocular torsion.

Another EVS parameter whose effects remain unclear is electrode montage. This parameter is critical, as the electrode montage dictates which vestibular and brain structures the applied EVS current passes through (Truong et al., 2024, Truong et al., 2023). Simulations of EVS current flow patterns using a binaural montage suggest that current passes through bilateral vestibular systems as well as the cerebellum, pons, and medulla (Truong et al., 2024). In contrast, simulations of a mastoid-nape (sub-occipital) monoaural montage indicate that current predominantly passes through a single vestibular system as well as parts of the cerebellum and spinal cord (Truong et al., 2024). Whether these simulations reflect *in vivo* current flow patterns in the human vestibular system remains unknown. Nevertheless, these results highlight the importance of electrode montage in determining whether current can be effectively lateralised to stimulate one vestibular end organ in isolation. In clinical contexts, such lateralisation is essential for accurately assessing residual vestibular function on each side and interaural asymmetry. Despite this importance, it remains unclear how different monoaural montages influence EVS-evoked ocular torsion and asymmetry.

The effects of stimulus frequency on EVS-evoked torsion and asymmetry also remain unclear. Ocular torsion follows the frequency of a sinusoidally-varying current – an electrical stimulus that varies in time according to a sine wave – applied to the mastoid process (Mackenzie and Reynolds, 2018). Sinusoidal stimuli therefore facilitate identification of torsional responses even when they are severely attenuated by vestibular pathology (Mackenzie et al., 2019, Mackenzie et al., 2018). Moreover, multiple pathologies – such as Ménière’s disease (Hannigan et al., 2021, McCaslin et al., 2015), vestibular neuritis (Zellhuber et al., 2014), vestibular migraine (Sinan Yilmaz et al., 2021), and acute vertigo (Olivecrona et al., 2022) – are characterised by dissociations in vestibular function at lower frequencies (<0.5 Hz; assessed through caloric irrigation) versus higher frequencies (≥2 Hz; assessed through video head impulse tests). By analogy, applying EVS stimuli at different frequencies could support a clinical assessment that distinguishes between vestibular pathologies. However, the relative advantages of stimuli at 0.5 Hz versus 2 Hz remain uncharacterised. For instance, previous work showed that delivering EVS at 0.5 Hz evokes larger ocular torsion than at 2 Hz (Mackenzie and Reynolds, 2018). Analogous to the ice-water caloric test (Batuecas-Caletrio et al., 2009), low-frequency stimuli may therefore be better suited for assessing residual vestibular function. However, whilst EVS stimuli at 2 Hz evoke smaller ocular torsion (Mackenzie and Reynolds, 2018), they produce more cycles per unit of time, potentially accelerating the assessment of interaural asymmetry. Characterising this trade-off between response amplitude and assessment speed for interaural asymmetry becomes especially relevant in time-constrained clinical contexts. Clarifying how stimulus frequency shapes this trade-off is therefore essential for optimising the clinical application of EVS.

The present study aimed to characterise the effects of ambient lights, electrode montage, and stimulus frequency on EVS-evoked ocular torsion. To investigate this, three experiments were carried out. The first experiment determined whether fixating on a light-emitting diode (LED) presented in ambient lights attenuates torsion relative to complete darkness. The second experiment investigated the effect of manipulating reference electrode placement upon torsion, with the aim of identifying an optimal montage for monaural vestibular stimulation. The third experiment characterised the effects of delivering sinusoidal EVS stimuli at 0.5 Hz, 2 Hz, and as average-of-sine stimuli on torsion. In all experiments, interaural asymmetry was also assessed.

## 2 Methods

### 2.1 Participants

A total of 53 neurologically and neuro-otologically healthy young adults took part in this work (mean age ± SD: 21.1 ± 3.2 years old; 26 females). This project was approved by the local institutional review board (project #MRC2023_01) and conformed to the Declaration of Helsinki. All participants provided informed written consent. A total of 72 experimental sessions over three distinct experiments were collected (24 sessions per experiment). Thirty-nine participants took part in a single experiment (39 sessions), nine participants took part in two experiments (18 sessions), and five participants took part in all three experiments (15 sessions). None of the participants took part more than once in each experiment.

For each experiment, the following experimental conditions were fully counterbalanced across participants: 3 EVS Parameter (varying between experiments) X 2 Stimulation Side (right, left) X 2 Current Intensity (2 mA, 4 mA). This resulted in a total of 24 possible condition orders (3! X 2! X 2!). To achieve full counterbalancing, 24 participants were recruited per experiment. To perform the analyses below, G*Power (v3.1.9.7; Faul et al., 2007) was used. G*Power is a freely available statistical tool to calculate required sample sizes and the statistical power a given sample size achieved, amongst its many other uses (Faul et al., 2007). A sensitivity analysis revealed that the smallest effect size that can be detected with 24 participants is a Cohen’s dz of 0.597 (moderate to large effect size), assuming 80 % power, a significance threshold (alpha) of 0.05 and two-tailed dependent t-tests. Similarly, a post-hoc power analysis revealed that groups of 24 participants could detect a Cohen’s dz of 0.8 (large effect size) with 96.3 % power, assuming a significance threshold (alpha) of 0.05 and two-tailed dependent t-tests. Taken together, these results suggest that the present design was adequately powered to detect medium to above-large within-subject effect sizes.

### 2.2 Devices

An isolated bipolar constant current stimulator (DS5, Digitimer, Letchworth Garden City, UK) was used to deliver EVS. The alternating current was delivered through carbon–rubber stimulation electrodes (46 x 37 mm) held with an adhesive dressing and prepared with 3 mL of electrolyte gel (Signa Gel, Parker Laboratories, Fairfield, NJ, USA). Because an alternating current was used, the polarity of the electrodes reversed continuously throughout each cycle. To standardise placement, the electrode serving as the anode during the first half-cycle of stimulation was consistently attached to the mastoid process. The alternating current was driven by a custom-made MATLAB script (R2022b, Mathworks ©) and controlled through a National Instrument board (model PCIe-6321; National Instruments Corporation, Austin, Texas, US). Tri-axial eye position data (torsional, horizontal, and vertical) from the right and left eyes were non-invasively recorded at 101 Hz using commercially available infrared camera-mounted goggles (Videonystagmography goggles, Interacoustics, Middelfart, Denmark; light wavelength of 940 nm). Tri-axial eye data were sampled using the Ocular Counter Roll software module, which is part of the MicroMedical VisualEyes software suite (Interacoustics, Middelfart, Denmark). This software estimates ocular torsion by tracking of iris features (Otero-Millan et al., 2015). The manufacturer’s calibration procedures for torsional and translational (horizontal and vertical) eye responses were implemented before collecting data. The luminance of the LED and ambient lights was assessed using a digital lux meter (model LX1330B, Dr. Meter ©).

### 2.3 Working around the lack of synchronisation between EVS and the eye data

The devices and software used did not allow for precise synchronisation between the EVS sinusoidal stimulus and the recording of tri-axial eye data. Two strategies were implemented to mitigate this. First, repeating sinusoidal EVS stimuli were used, evoking torsion at the stimulus frequency (as in Mackenzie and Reynolds, 2018; see Fig. 4, Fig. 7, Fig. 9 as well as Supplementary Figs. 1–10 for posthoc support). By utilising a repeating sinusoidal stimulus, the amplitude of the resulting frequency-specific torsion could be readily identified and calculated.

Second, for each trial, tri-axial eye data were recorded for 60 s, whereas the EVS stimulus was delivered for 50 s. To account for the uncertainty in the timing of EVS stimulus onset and offset, the first and last 10 s of eye data were systematically discarded. This yielded 40-second segments centred on the period of active stimulation, which were used for analyses. Post-hoc results supported the adequacy of these 40-segment segments for reliably estimating torsion and asymmetry (see Fig. 12, Fig. 13). Additionally, discarding the first and last 10 s of each segment protected against bandpass-filtering-induced edge artefacts (see Section 2.5). For graphical display of averaged response cycles (Fig. 4, Fig. 7, Fig. 9), the first peak of ocular torsion per cycle (one full cycle at 0.5 Hz; first of four cycles at 2 Hz) were segmented, aligned, and averaged. This resulted in the averaging of twenty 2-second segments across each 40-second segment.

### 2.4 Overall protocol

Hereafter, for conciseness and readability, the term “torsion” refers to “ocular torsion” and “asymmetry” refers to “interaural vestibular asymmetry”. Despite testing different hypotheses, each experiment delivered a current of ± 2 mA and ± 4 mA to the right and left mastoid processes using a monoaural montage (except in EXP #3, where a binaural montage was also used). The current of ± 2 mA and ± 4 mA implies that the peak-to-peak current was of 4 mA and 8 mA, respectively. For conciseness, the “±” sign is hereafter omitted when referring to current intensity in the text. The separate activation of the right and left vestibular systems was necessary to assess asymmetry. Moreover, one condition set was repeated across all experiments: mastoid-C7 monoaural montage, LED fixation in darkness, and sines at 0.5 Hz delivered at 2 mA and 4 mA. This was implemented to assess the range of absolute torsional responses and asymmetry across a large sample (n = 53), thereby providing normative reference data (see Fig. 14).

#### 2.4.1 EXP #1 – Effect of ambient lights and LED fixation on EVS-evoked torsion

The first experiment tested two hypotheses. First, it was hypothesised that fixating on an LED would suppress EVS-evoked horizontal and vertical nystagmus, compared to fixating on an “imaginary LED” in darkness (Hirvonen et al., 2012, Korda et al., 2021). Second, it was also hypothesised that providing ambient lights simultaneously with LED fixation would attenuate the amplitude of EVS-evoked torsion compared to fixation in darkness (Straumann et al., 1992).

**Fig. 1:**
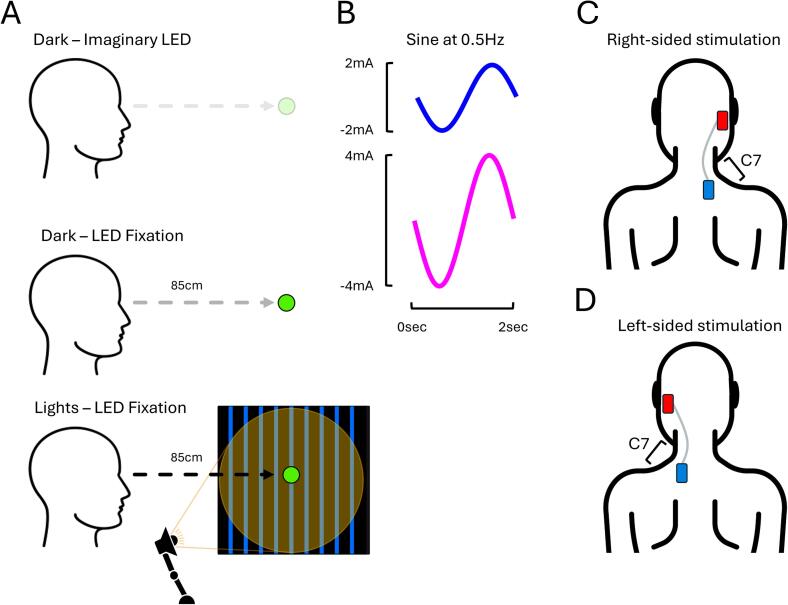
**Experimental conditions from EXP#1. (A)** Participants either fixated on an imaginary LED in darkness, a real LED in darkness, or a real LED presented in ambient lights. In this panel, the green-filled circle represents the LED used for fixation, and the black square depicts the earth-referenced visual background used to emphasise gravitational verticality when ambient lights were present. In the imaginary LED condition, the LED was not visible. **(B)** For all conditions, EVS was delivered as a sinusoidal stimulus at 0.5 Hz with a current intensity of either 2 mA or 4 mA. **(C–D)** A mastoid–C7 monoaural montage delivered current either through the right (C) or left mastoid process **(D).**

To test these hypotheses, participants either fixated on an imaginary LED in complete darkness (0 lx), a real LED (3 lx) in darkness (0 lx background), or a real LED (3 lx) presented in front of a vertically striated visual scene illuminated by ambient light (250 lx; Fig. 1A). In the “imaginary LED” condition, participants were instructed to imagine fixating on a small point of light in complete darkness, located at the same position as the real LED used in the other conditions. Hereafter, these conditions are respectively referred to as “Dark – Imaginary LED”, “Dark – LED Fixation”, and “Lights – LED Fixation”. In the Lights – LED Fixation condition, a vertically striated visual scenery was used to provide participants a stable visual reference frame aligning with the earth’s gravitational plane. The scene contained nine bright-blue-coloured vertical striations, each separated by 15 cm and presented on a black background. In all three conditions, a sinusoidal stimulus at 0.5 Hz was delivered at either 2 mA or 4 mA (Fig. 1B) with a mastoid-C7 monoaural montage (Fig. 1C and 1D).

#### 2.4.2 EXP#2 – Effect of electrode montage on EVS-evoked torsion

The second experiment tested whether different monoaural montages could isolate the activation of a single vestibular end organ. Specifically, it was hypothesised that placing the reference electrode further away from the “unstimulated” mastoid would reduce unwanted current spread to that side. If correct, this would yield a floor effect in torsion amplitude – that is, a measurable point at which torsion no longer decreases despite further attempts to reduce contralateral stimulation – indicating minimal activation of the contralateral vestibular end organ. Such a monoaural montage would be ideal for assessing asymmetry. Conversely, if no floor effect emerges, this would suggest that all monoaural montages result in contralateral current spread, limiting their utility for assessing asymmetry.

**Fig. 2:**
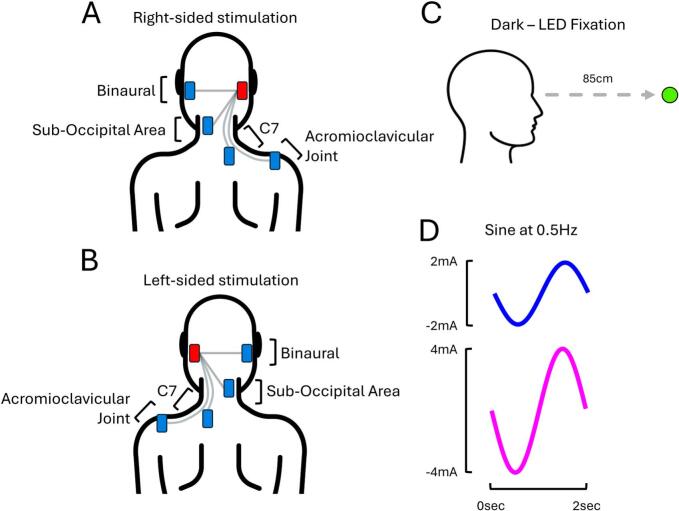
**Experimental conditions from EXP#2.** When electrode montages were monoaural, the stimulating electrode was either positioned over the right **(A)** or left **(B)** mastoid process. **(C)** Participants systematically fixated on an LED in complete darkness. **(D)** For all montages, EVS was delivered as a sinusoidal stimulus at 0.5 Hz with a current intensity of either 2 mA or 4 mA.

To investigate this, EVS was delivered using four electrode montages: one binaural and three monoaural (Fig. 2A and B). The binaural montage delivered current between the two mastoids and served as a positive control, under the assumption that activation of both mastoids is maximal. This was used to determine whether, in monoaural montages, torsion amplitude decreases as a function of the distance between the reference electrode and the “unstimulated” mastoid. The binaural conditions were delivered at 2 mA then 4 mA, and were systematically collected before the monoaural ones.

For all monoaural montages, one electrode was systematically placed on the “stimulated” right or left mastoid, whilst the location of the reference electrode was manipulated. It was either positioned on the “Sub-Occipital Area” (5 cm lateral to the neck’s midline, contralateral to the stimulated mastoid), next to C7 (5 cm lateral to the neck’s midline, ipsilateral to the stimulated mastoid), or on the acromioclavicular joint (ipsilateral to the stimulated mastoid). Hereafter, these conditions are respectively referred to as “Sub-Occipital area”, “C7”, and “Acromioclavicular joint”. Although current flow patterns during EVS remain poorly understood in humans (Truong et al., 2024), these electrode locations were chosen to create a systematic gradient in distance and orientation (see Fig. 2A and B). This was expected to progressively divert current away from the “unstimulated” mastoid, thereby selectively activating one vestibular system. The average radial distance (±SD) on the skin that separated the reference electrode’s centroid and the unstimulated mastoid was 8.2 ± 0.9 cm, 17.2 ± 1.0 cm and 32.7 ± 2.6 cm for the Sub-Occipital area, C7 and Acromioclavicular joint montages, respectively. Finally, for all four montages, participants fixated on an LED (3 lx) in complete darkness (Dark – LED Fixation; Fig. 2C) whilst sines at 0.5 Hz were delivered at either 2 mA or 4 mA (Fig. 2D).

#### 2.4.3 EXP#3 – Effects of different sinusoidal EVS stimuli on torsion

The third experiment investigated how the frequency of sinusoidal (sine) EVS stimuli influences torsion and asymmetry. It was expected that sine stimuli at 0.5 Hz would evoke greater torsion than at 2 Hz (Mackenzie and Reynolds, 2018). It was also expected that assessing asymmetry using 2 Hz sine stimuli would require a shorter stimulation duration than at 0.5 Hz because more cycles of torsion are evoked per unit of time. Whether combining sines at 0.5 Hz and 2 Hz into average-of-sine stimuli would allow efficient estimation of torsion and asymmetry within a single trial was also explored.

**Fig. 3:**
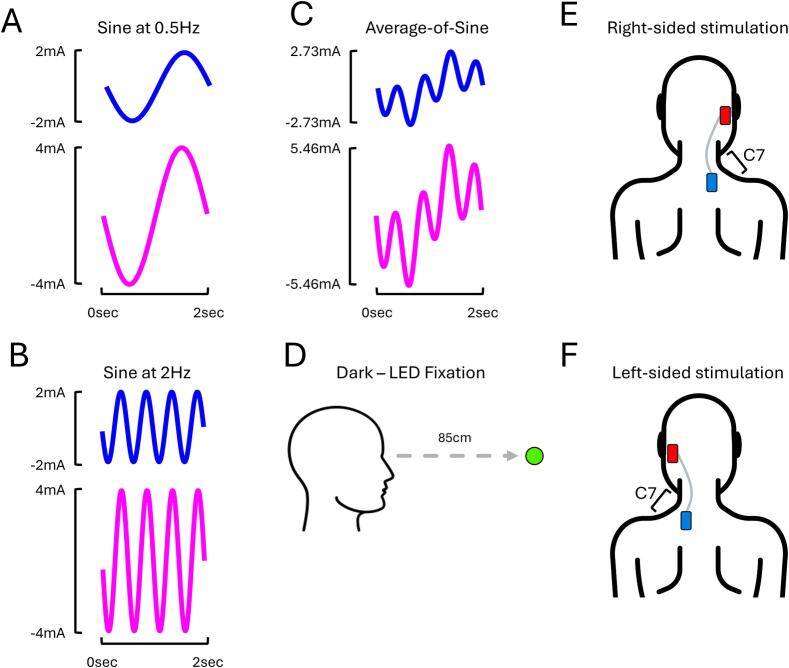
**Experimental conditions from EXP#3.** Visual depiction of a 2-second-long segment of the sinusoidal stimuli at 0.5 Hz **(A)**, 2 Hz **(B)**, and the stimulus resulting from summing the 0.5 Hz and 2 Hz stimuli **(C)**. **(D)** Participants systematically fixated on an LED in darkness. A mastoid-C7 monoaural montage delivered a current intensity of either 2 mA or 4 mA through the right **(E)** or left mastoid process **(F)**.

To investigate this, three sinusoidal stimuli were delivered: sines at 0.5 Hz (Fig. 3A), sines at 2 Hz (Fig. 3B), and an average-of-sine stimuli combining sines at 0.5 Hz and 2 Hz into a single stimulus (Fig. 3C). This composite waveform was created by averaging the full-amplitude 0.5 Hz and 2 Hz sine waves without phase offset, then normalised to match the root mean square (RMS) amplitude of the individual sine waveforms. RMS normalisation reduced the amplitude of each frequency component by a factor of 1/√2 (∼0.71), corresponding to a ∼ 29 % decrease relative to the individual sine stimuli, ensuring equivalent net energy levels over time. This procedure isolated the effects of waveform composition – rather than stimulus intensity – on vestibular afferent activation and torsional eye responses. Normalisation also helped to minimise discomfort and prevent excessive peak currents from direct summation (3.86 mA and 7.71 mA for combined sine components with 2 mA and 4 mA amplitudes, respectively), which could have recruited additional afferents beyond those activated by the 2 mA and 4 mA stimuli alone. When energy-matched to the 2 mA and 4 mA sines, the average-of-sine waveform had peak currents of 2.73 mA and 5.46 mA, respectively. Hereafter, these conditions are referred to as “Sine at 0.5 Hz”, “Sine at 2 Hz”, and “Average-of-Sine”. Participants fixated on an LED in darkness (Dark – LED Fixation; Fig. 3D), whilst EVS stimuli were delivered at either 2 mA or 4 mA (2.73 mA or 5.46 mA for the average-of-sine stimuli) with a mastoid-C7 montage (Fig. 3E and F).

One caveat of summing the two sine stimuli at full amplitude without phase lags is that the resulting current intensities are approximately 36 % greater (Fig. 3C). This increase in current intensity was expected to increase participants’ discomfort on the skin. To investigate this, participants were asked to rate their perceived level of overall discomfort immediately after each stimulation trial using a visual analogue scale ranging from 0 (no discomfort) to 10 (extreme discomfort; see Fig. 11).

To assess how the three sinusoidal stimuli performed in assessing asymmetry, additional analyses were conducted to determine the segment duration of torsion data (in seconds) required to obtain a stable measure of asymmetry. This was achieved by recalculating asymmetry iteratively, increasing the segment duration of torsion in 2-second increments up to 40 s. To determine when the asymmetry measure stabilised, the difference between successive segments (e.g., 4 sec vs 2 sec, 6 sec vs 4 sec, 8 sec vs 6 sec, etc.) was calculated. The stabilisation of asymmetry was defined as a mean difference of 0 ± 2.5 % between segments – that is, when asymmetry fluctuated less than 5 % around a consistent value. These procedures were applied separately to the 0.5 Hz, 2 Hz, and average-of-sine stimuli. The results are shown **in** Fig. 12, Fig. 13.

### 2.5 Data acquisition and processing

**Fig. 4:**
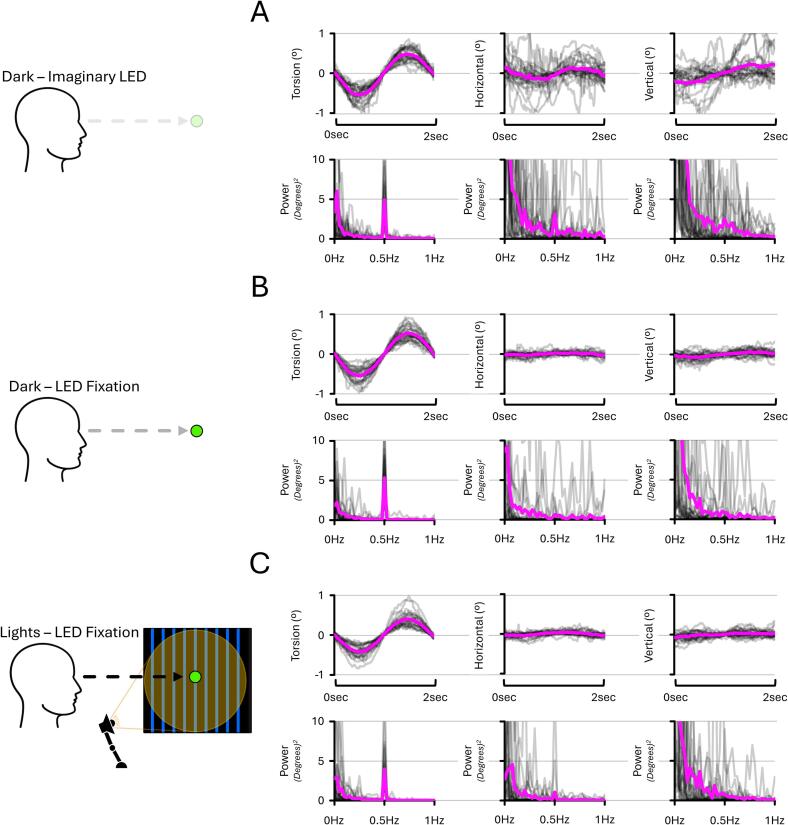
**Torsional, horizontal, and vertical eye responses and corresponding Fourier spectra from EXP#1.** Panels show responses under three light conditions: imaginary LED **(A)**, LED fixation in darkness **(B)**, and LED fixation with ambient lights **(C)**, all during 4 mA right-sided stimulation with a mastoid-C7 montage. LED fixation suppressed horizontal and vertical responses at 0.5 Hz. Grey lines show individual data; the magenta line indicates the group mean. Positive values reflect counter-clockwise (torsion), rightward (horizontal), and upward (vertical) eye responses.

For each condition and experiment, participants were instructed to keep their eyes as wide open as possible and to blink as little as possible to enable stable eye tracking during the 60-second segments. Data from both eyes were acquired and processed as follows. First, the sampling rate of the data was resampled to 100 Hz. Second, eyeblinks were detected using the “hampel” function in MATLAB, which acts as a filter detecting outliers based on median absolute deviations (MADs). The MADs were calculated over 2-second segments (200 samples), coinciding with a full cycle of torsion when delivering EVS stimuli at 0.5 Hz. Third, a third-order one-dimensional median filter (“medfilt1” function in MATLAB) was applied to remove the remaining outlying points. This did not distort the eye responses and was implemented to ensure that no outlier remained, which could confound the ensuing data reconstruction. Fourth, the signal was reconstructed using the function “fillgaps” in MATLAB. This function fills the gaps in missing data by using autoregressive models that minimise the Akaike information criterion. Hereafter, the data that has been gap-filled but not bandpass-filtered is referred to as “reconstructed data”. Importantly, this step was essential to obtain a continuous time series suitable for subsequent frequency-domain analyses (i.e., bandpass filtering and fast Fourier transform). Examples of 2-second segments of reconstructed eye data are presented in Fig. 4, Fig. 7, Fig. 9, whereas 2-second segments of reconstructed eye data for each condition are presented in Supplementary Figs. 1, 3, 5, 7, and 9. Whilst torsional and horizontal responses to EVS are oriented in the same direction for both eyes, the vertical responses are known to be disconjugate (Severac et al., 2003). Hence, for the purposes of graphical representation (Fig. 4, Fig. 7, Fig. 9**)**, vertical responses from the right eye were inverted to match those of the left eye.

To inform data processing in the frequency domain, one-sided fast Fourier transforms (“fft” function in MATLAB) were performed on the reconstructed torsion, horizontal and vertical data of the 40-second segments (4000 samples) coinciding with EVS delivery. Examples of the resulting power-frequency spectra are shown in Fig. 4, Fig. 7, Fig. 9, whereas the power-frequency spectra of each condition are presented in Supplementary Figs. 2, 4, 6, 8, and 10. Visual inspection revealed overt frequency components that matched the sinusoidal EVS stimuli frequency (0.5 Hz and/or 2 Hz) in the torsion data, whereas noisy and unsystematic components at these frequencies emerged in the horizontal and vertical data. As such, further processing in the frequency domain was only applied to torsion. However, although embedded in noise, the presence of responses at 0.5 Hz cannot be fully excluded for horizontal and vertical data when participants did not fixate on an LED (Dark – Imaginary LED condition of EXP#1; see Fig. 4 and Supplementary Figs. 3–6).

For torsion only, narrow bandpass filters matching the frequency of the sinusoidal EVS stimulus were applied to the 60-second segments. Specifically, a bandpass filter of 0.4 to 0.6 Hz and 1.9 to 2.1 Hz were applied when sines at 0.5 Hz and 2 Hz were delivered, respectively. For the average-of-sine stimuli, the time-course of torsion was duplicated and bandpass filtered separately: one time-course was filtered from 0.4 to 0.6 Hz, and the other from 1.9 and 2.1 Hz. Then, the peak-to-peak amplitude of the resulting signal was calculated for each cycle of the 40-second segments coinciding with EVS delivery. This resulted in 20 and 80 amplitude values for signal bandpass-filtered at 0.5 Hz and 2 Hz, respectively. Concerning translational responses, standard deviations (SD) were calculated over 2-second segments to assess the variability of the reconstructed horizontal and vertical data. This resulted in 20 SD values for each 40-second segment. Although the data from both eyes were processed as described above, the eye that produced the largest average torsional eye responses was selected to quantify the dependent variables (see below).

### 2.6 Dependent variables

The following dependent variables were measured separately for each condition and experiment. Torsion was quantified in degrees (°) by averaging the peak-to-peak amplitude values from each cycle of the 40-second segments of bandpass-filtered torsion data. Note that torsion amplitudes are consistently expressed as peak-to-peak measures throughout. To quantify asymmetry in torsion data, the Jongkees’ formula was used (Alhabib and Saliba, 2022, Jongkees et al., 1962): ((Right-Left)/(Right + Left)) × 100. This resulted in a signed percentage value (%), where positive and negative values indicate unilateral weakness of the left and right sides, respectively. Despite criticisms (Furman and Jacob, 1993), the Jongkees’ formula was implemented because it remains the standard approach to calculating asymmetry in clinical practice (i.e., when a caloric irrigation test is performed). Therefore, it was reasoned that implementing the Jongkees formula would increase the clinical relevance of EVS-evoked asymmetry assessments. Finally, the variability of horizontal and vertical responses was quantified in degrees (°) by averaging the 20 SD values.

### 2.7 Statistical analyses

Three-way repeated measure analyses of variance (rmANOVAs) were conducted to analyse the data. The Greenhouse-Geisser correction was implemented when violations of sphericity occurred (*p* < 0.05; Mauchly’s test). All experiments had three within-subject fixed effects. Of those three fixed effects, two were present in each experiment: 2 Current Intensity (2 mA, 4 mA) X 2 Stimulation Side (Right, Left). Here and throughout, the “X” denotes an interaction term between fixed effects. In EXP#1, the additional fixed effect was 3 Light Condition (Dark – Imaginary LED, Dark – LED Fixation, Lights – LED Fixation). In EXP#2, the additional fixed effect was 3 Electrode Location (Sub-Occipital Area, C7, Acromioclavicular joint). In EXP#3, the additional fixed effect was 3 EVS Stimulus (Sine at 0.5 Hz, Sine at 2 Hz, Average-of-Sine). For pairwise comparisons, paired-sample t-tests or Wilcoxon’s tests if data violated normality (*p* < 0.05; Shapiro-Wilk’s test) were conducted. To correct for multiple comparisons, the Benjamini-Hochberg (Benjamini and Hochberg, 1995) correction was implemented. P values < 0.05 were deemed statistically significant. These analyses were conducted using JAMOVI (Sahin and Aybek, 2020)(v2.3.28). Unless otherwise specified, reported statistics and error bars in the figures hereafter represent the mean ± 95 % CIs. Note that these CIs are not directly indicative of individual variability and should not be used to determine clinical normality thresholds. Cohen’s dz and $\eta_{p}^{2}$ are reported as effect sizes for pairwise comparions and rmANOVAs, respectively (see Lakens, 2013). For conciseness, “dz” is hereafter used to refer to “Cohen’s dz”.

## 3 Results

### 3.1 EXP#1 – Effects of light

Fig. 4 illustrates that EVS stimuli at 0.5 Hz evoked a systematic 0.5 Hz component in the torsion data. In contrast, similar 0.5 Hz components were not systematically present in the horizontal and vertical data and were instead embedded in non-negligible noise. This suggests that torsional responses are strongly frequency-specific when delivering EVS stimuli at 0.5 Hz, whereas horizontal and vertical responses are not. See Supplementary Figs. 1–6 for full report.

#### 3.1.1 Fixating on an LED with ambient lights decreased torsion amplitude by 20 to 25 %

**Fig. 5:**
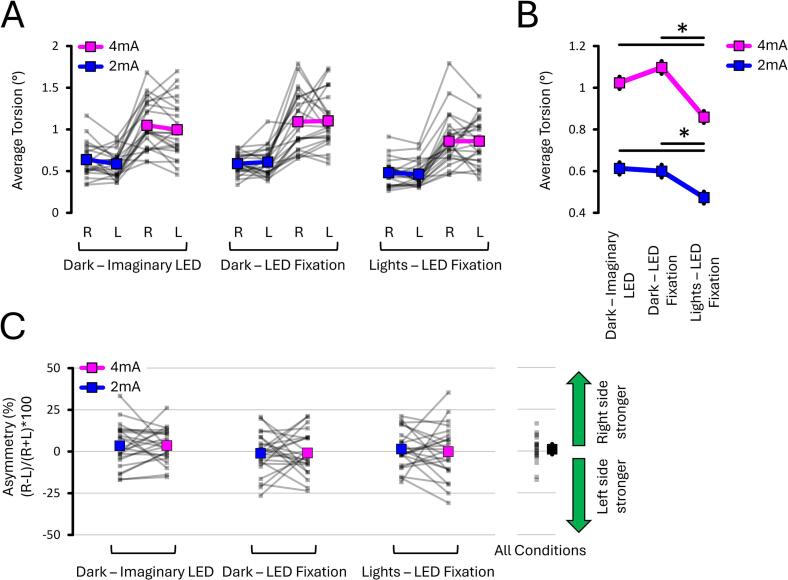
**Torsion and asymmetry results from EXP#1.** Torsion amplitude **(A, B)** and asymmetry **(C)** across fixation and lighting conditions as well as current intensities. Torsion was attenuated by ambient lights. Mean asymmetry did not differ between conditions and remained near zero. Grey lines show individual data; bold coloured lines and markers indicate condition means. Asterisks indicate significant differences (p < 0.05). “R” and “L” = right- and left-sided stimulation, respectively.

The torsion data (Fig. 5A) revealed a Light Condition X Current Intensity interaction (*p* = 0.048, $\eta_{p}^{2}$ = 0.123). This further revealed that, at 2 mA (Fig. 5B), torsion for Dark – Imaginary LED (0.61 ± 0.03°) did not differ from Dark – LED Fixation (0.60 ± 0.03°; *p* = 0.624, dz = 0.102). However, torsion on both these conditions was 25 % greater than Lights – LED Fixation (0.47 ± 0.03°; both *p* < 0.001, both dz > 0.993). Similarly, at 4 mA (Fig. 5B), torsion for Dark – Imaginary LED (1.02 ± 0.07°) did not differ from Dark – LED Fixation (1.10 ± 0.05°; *p* = 0.209, dz = -0.264). Importantly, torsion on both these conditions was 20 to 25 % greater than Lights – LED fixation (0.86 ± 0.06°; both *p* < 0.023, both dz < 0.561). Overall, this shows that ambient lights decrease torsion amplitude compared to darkness. Moreover, the results suggest that fixating on either an imaginary or a real LED in darkness does not significantly alter torsion.

#### 3.1.2 Asymmetry was not influenced by ambient lights or current intensity (2 mA, 4 mA)

The asymmetry data (Fig. 5C) revealed no Light Condition X Current Intensity interaction (*p* = 0.920, $\eta_{p}^{2}$ = 0.004), no effect of Light Condition (*p* = 0.112, $\eta_{p}^{2}$ = 0.091), and no effect of Current Intensity (*p* = 0.824, $\eta_{p}^{2}$ = 0.002). Moreover, the mean asymmetry of each condition did not differ from zero (all *p* > 0.545, all dz < 0.360). Across all conditions (rightmost panel of Fig. 5C), asymmetry averaged 1.09 % ± 3.06 % (min: −17.11 %; max: 16.58 %). This suggests asymmetry was not influenced by LED fixation, ambient lights or current intensity.

#### 3.1.3 Fixating on an LED attenuated the variability of horizontal and vertical eye responses

**Fig. 6:**
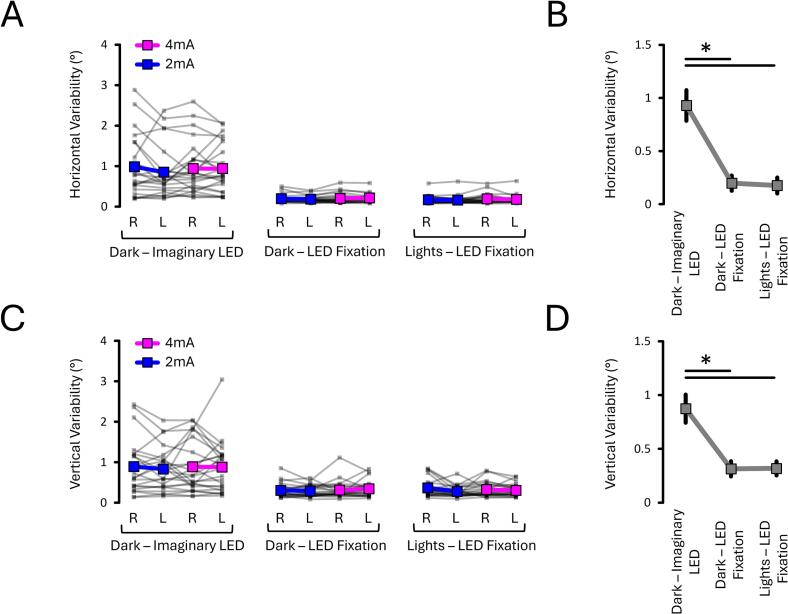
**Variability of horizontal (A–B) and vertical (C–D) eye responses from EXP#1.** Response variability was greatest without LED fixation (“Imaginary LED” condition) and lowest during LED fixation, regardless of ambient lights. Grey lines show individual data; bold coloured and grey lines indicate condition means. Asterisks (*) indicate significant differences (p < 0.05). “R” and “L” = right- and left-sided stimulation, respectively.

The horizontal data (Fig. 6A and B) selectively revealed an effect of Light Condition (*p* < 0.001, $\eta_{p}^{2}$ = 0.828). This further revealed that the variability of horizontal responses was greater in the Dark – Imaginary LED (0.93 ± 0.14°) compared to both Dark – LED Fixation (0.20 ± 0.07°; *p* < 0.001, dz = 1.360) and Lights – LED Fixation (0.18 ± 0.07°; *p* < 0.001, dz = 1.376). The variability of horizontal responses in Lights – LED Fixation did not differ from those in the Dark – LED Fixation (0.18 ± 0.07°; *p* = 0.792, dz = -0.054). Overall, this shows that fixating on an LED attenuated the variability of horizontal responses regardless of ambient lights.

The vertical data (Fig. 6C and D) also revealed an effect of Light Condition (*p* < 0.001, $\eta_{p}^{2}$ = 0.568). As with the horizontal responses, the variability of vertical responses was greater in the Dark – Imaginary LED (0.87 ± 0.13°) compared to both Dark – LED Fixation (0.31 ± 0.07°; *p* < 0.001, dz = 1.212) and Lights – LED Fixation (0.32 ± 0.07°; *p* < 0.001, dz = 1.145). The variability of vertical responses did not differ between Dark – LED Fixation and Lights – LED Fixation (*p* = 0.317, dz = -0.041). Overall, this shows that fixating on an LED attenuated the variability of vertical responses regardless of ambient lights.

### 3.2 EXP#2 – Effects of electrode montage

**Fig. 7:**
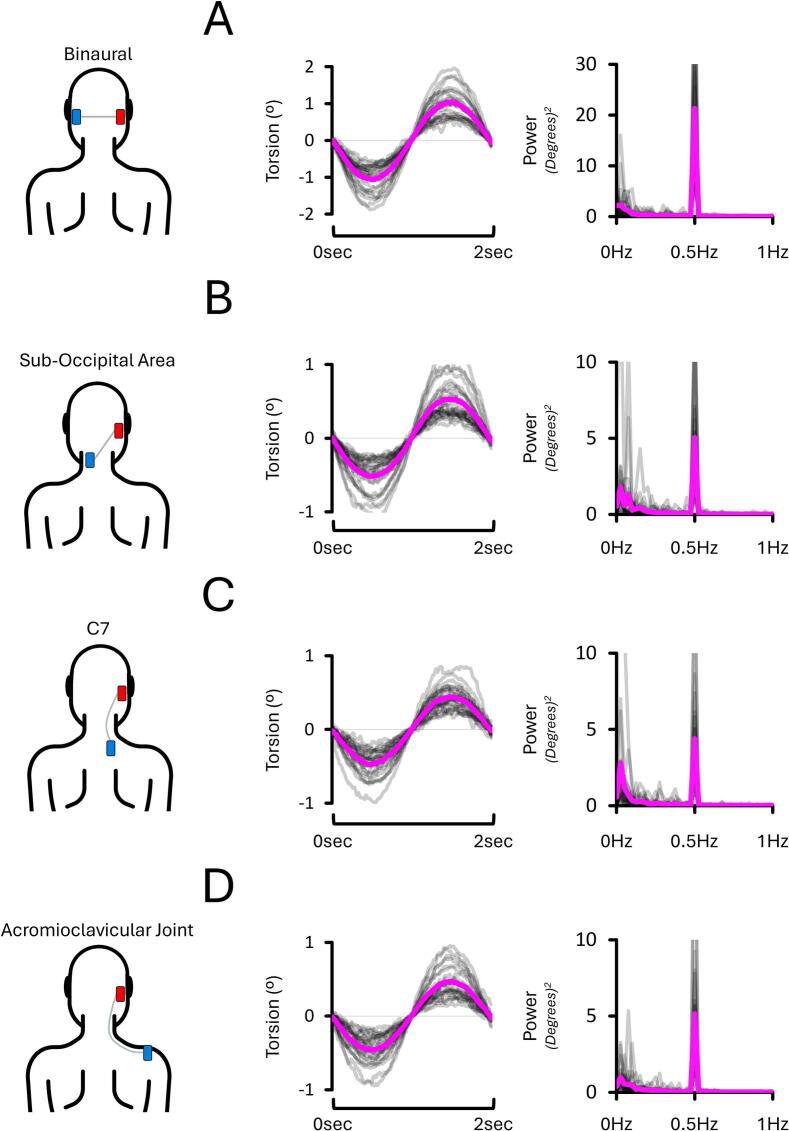
**Torsional eye responses and corresponding Fourier spectra from EXP#2**. Panels show torsional responses under four electrode montages: binaural **(A),** mastoid–suboccipital **(B),** mastoid–C7 **(C),** and mastoid–acromioclavicular joint **(D**), all during 4 mA right-sided stimulation with LED fixation in darkness. Monoaural montages decreased torsion compared to binaural stimulation. Note that the Y-axis scales are larger in panel **(A)** to accommodate the greater response amplitude. Grey lines show individual data; the magenta line indicates the condition mean. Positive values reflect clockwise torsion from the participant’s perspective.

Fig. 7 illustrates that EVS stimuli at 0.5 Hz evoked a systematic 0.5 Hz component in the torsion data, regardless of the electrode montage. See Supplementary Figs. 7 and 8 for a full report.

#### 3.2.1 Monoaural montages decreased torsion by 50 % compared to a binaural one

**Fig. 8:**
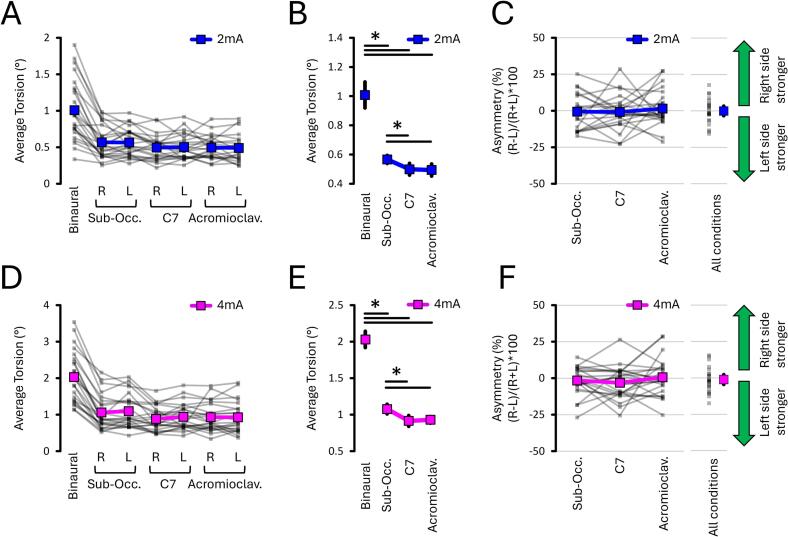
**Torsion and asymmetry results from EXP#2.** Panels show torsion amplitude across electrode montages **(A, D),** relative reductions under monoaural stimulation **(B, E),** and asymmetry measures **(C, F**) at 2 mA and 4 mA. The mastoid–suboccipital montage evoked slightly greater torsion than other monoaural montages. Mean asymmetry did not differ across conditions and remained near zero. Grey lines show individual data; bold coloured lines indicate condition means. Asterisks (*) denote significant differences (p < 0.05). “R” and “L” = right- and left-sided stimulation, respectively.

The torsion data (Fig. 8A-B-D-E) revealed that monoaural montages, regardless of the reference electrode location and side of the stimulation, decreased torsion by approximately 50 % compared to a binaural montage; this was the case for both 2 mA (all *p* < 0.001, all dz > 1.506) and 4 mA conditions (all *p* < 0.001, all dz > 2.304). Overall, this shows that torsion evoked by monoaural montages is approximately half of those evoked by a binaural one. By the same token, this suggests that monoaural montages redirect most of the EVS current away from the “unstimulated” mastoid.

#### 3.2.2 Torsion reached a floor effect when using a mastoid-C7 montage

At 2 mA, the torsion data (Fig. 8A and B) selectively revealed an effect of Electrode Location (*p* < 0.001, $\eta_{p}^{2}$ = 0.319). This further revealed that torsion was greater when the reference electrode was positioned on the contralateral sub-occipital area (0.56 ± 0.03°) compared to the ipsilateral side next to C7 (0.50 ± 0.04°; *p* = 0.004, dz = 0.684) or the acromioclavicular joint (0.49 ± 0.04°; *p* = 0.003, dz = 0.723). When the reference electrode was positioned next to C7, torsion did not differ compared to the acromioclavicular joint (*p* = 0.755, dz = 0.064). This suggests a floor effect was reached with the mastoid-C7 montage at 2 mA.

Similar results were revealed at 4 mA. Namely, the torsion data (Fig. 8D and E) also selectively revealed an effect of Electrode Location (*p* = 0.004, $\eta_{p}^{2}$ = 0.251). This showed that torsion was also greater when the reference electrode was positioned on the contralateral sub-occipital area (1.08 ± 0.07°) compared to the ipsilateral side next to C7 (0.91 ± 0.07°; *p* = 0.006, dz = 0.583) or the acromioclavicular joint (0.93 ± 0.05°; *p* = 0.002, dz = 0.765). When the reference electrode was positioned next to C7, torsion did not differ compared to the acromioclavicular joint (*p* = 0.663, dz = -0.085). This suggests a floor effect was reached with the mastoid-C7 montage at 4 mA.

#### 3.2.3 Asymmetry was not influenced by monoaural electrode montages

The asymmetry data (Fig. 8C–F) revealed no effect of Electrode Location X Current Intensity interaction (*p* = 0.932, $\eta_{p}^{2}$ = 0.003), no effect of Electrode Location (*p* = 0.268, $\eta_{p}^{2}$ = 0.055), and no effect of Current Intensity (*p* = 0.491, $\eta_{p}^{2}$ = 0.021). Moreover, the mean asymmetry of each condition did not differ from the zero (all *p* > 0.812, all dz < 0.280). Across all reference electrode locations and current intensities, the asymmetry averaged −0.69 % ± 2.75 % (min: −15.34 %; max: 12.50 %). Overall, this shows that asymmetry was not influenced by the electrode montage and current intensity.

### 3.3 EXP#3 – Effects of EVS stimuli frequency

**Fig. 9:**
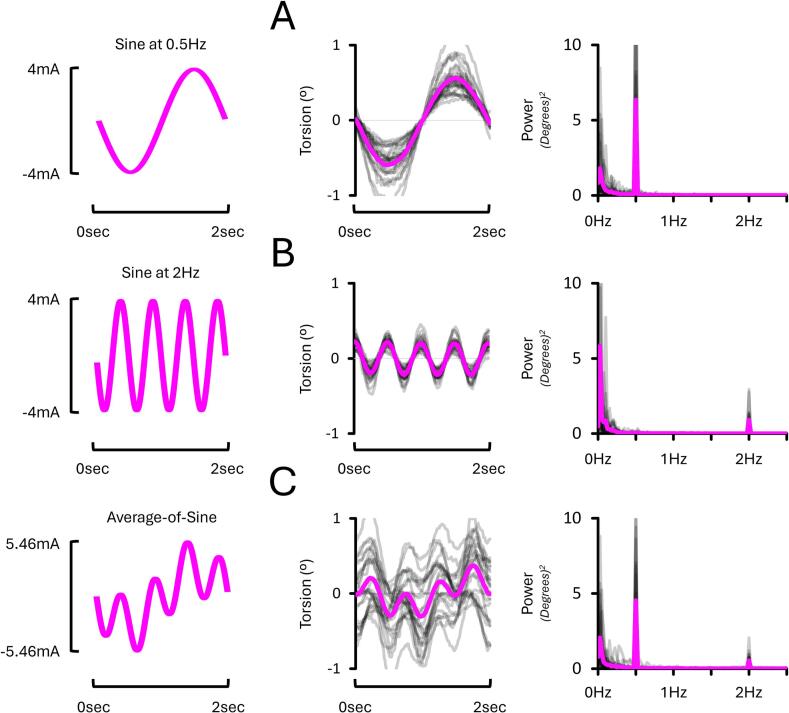
**Torsional eye responses and corresponding Fourier spectra from EXP#3.** Panels show responses to sines at 0.5 Hz **(A)**, 2 Hz **(B)**, and average-of-sine stimuli **(C)**, all during 4 mA right-sided stimulation with LED fixation in darkness and mastoid-C7 montage. Note that the average-of-sine stimuli combined two full-amplitude sine components, resulting in a peak current of 5.46 mA. All stimuli evoked frequency-specific components in torsional responses. Grey lines show individual data; the magenta line indicates the group mean. Positive values reflect clockwise torsion from the participant’s perspective.

Fig. 9 illustrates that EVS stimuli at 0.5 Hz and 2 Hz evoked systematic 0.5 Hz and 2 Hz frequency components in torsion data, respectively. Moreover, delivering average-of-sine stimuli simultaneously evoked frequency components at both 0.5 Hz and 2 Hz. See Supplementary Figs. 9 and 10 for a full report.

#### 3.3.1 Sines at 0.5 Hz evoked torsion 250 to 275 % greater than sines at 2 Hz

**Fig. 10:**
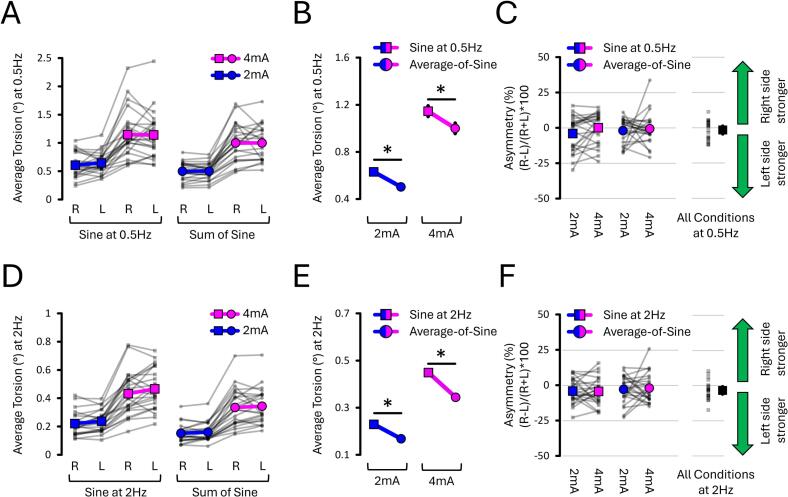
**Torsion and asymmetry results from EXP#3.** Panels show torsion amplitude at 0.5 Hz **(A),** a relative reduction with average-of-sine stimuli **(B),** and asymmetry at 0.5 Hz **(C)**; equivalent measures for 2 Hz stimuli are shown in **(D–F).** Average-of-sine decreased torsion amplitude compared to pure single-frequency stimuli. Mean asymmetry did not differ between conditions and remained near zero. Grey lines show individual data; bold coloured lines indicate condition means. Asterisks (*) denote significant differences (p < 0.05). “R” and “L” = right- and left-sided stimulation, respectively.

In the following analyses, torsion amplitude was compared between sines at 0.5 Hz and 2 Hz. The average-of-sine data are not included in these analyses. The torsion data (Fig. 9 and 10A-B-D-E) revealed an EVS Stimulus X Current Intensity interaction (*p* < 0.001, $\eta_{p}^{2}$ = 0.685). To decompose this interaction, torsion amplitude was analysed separately for each level of current intensity (2 mA, 4 mA); condition-specific means are reported below. At 2 mA, torsion evoked with sines at 0.5 Hz (0.63 ± 0.02°) was 275 % greater than those evoked with sines at 2 Hz (0.23 ± 0.01°; *p* < 0.001, dz = 2.601). Similarly, at 4 mA, torsion evoked at 0.5 Hz (1.14 ± 0.05°) was 250 % greater than those evoked with sines at 2 Hz (0.45 ± 0.01°; *p* < 0.001, dz = 2.155). Overall, this shows that sines at 0.5 Hz evoke greater torsion amplitude than at 2 Hz.

#### 3.3.2 Torsion at 0.5 Hz: Average-of-Sine vs pure 0.5 Hz sines

In the following analyses, torsion amplitude measured from the 0.5 Hz frequency components of the average-of-sine stimuli was compared to torsion evoked by “pure” sines at 0.5 Hz. As expected, torsion evoked from the 0.5 Hz component of the average-of-sines stimulus was smaller than that evoked by the pure 0.5 Hz sine. This is because, in the average-of-sines stimulus, the amplitude of each individual frequency component was reduced relative to the corresponding single-frequency stimulus.

The torsion data **(**Fig. 10A and B) selectively revealed an effect of EVS Stimulus (*p* = 0.001, $\eta_{p}^{2}$ = 0.415) and Current Intensity (*p* < 0.001, $\eta_{p}^{2}$ = 0.874). To assess the effect of EVS Stimulus, data from both levels of current intensity (2 mA, 4 mA) were combined. This revealed that torsion evoked from the pure sines at 0.5 Hz (0.89 ± 0.03°) was 20 % greater than those measured from the 0.5 Hz component of the average-of-sine stimuli (0.75 ± 0.03°; *p* = 0.001, dz = 0.841). Similarly, to assess the effect of Current Intensity, data from both EVS Stimulus (Sine at 0.5 Hz, 0.5 Hz component of the average-of-sine stimuli) were combined. As expected, this revealed that torsion at 0.5 Hz is 50 % smaller at 2 mA (0.56 ± 0.04°) compared to 4 mA (1.07 ± 0.04°; *p* < 0.001, dz = 2.553). Overall, this shows that torsion is 20 % greater when evoked by pure sines at 0.5 Hz, compared to the 0.5 Hz component of the average-of-sine stimuli, as expected due to the difference in stimulus magnitude.

#### 3.3.3 Asymmetry at 0.5 Hz was not influenced by EVS stimuli or current intensity

When measured from the bandpass-filtered 0.5 Hz frequency components, the asymmetry data (Fig. 10C) revealed no EVS Stimulus X Current Intensity interaction (*p* = 0.159, $\eta_{p}^{2}$ = 0.084), no effect of EVS Stimulus (*p* = 0.253, $\eta_{p}^{2}$ = 0.056), and no effect of Current Intensity (*p* = 0.212, $\eta_{p}^{2}$ = 0.067). Moreover, the mean asymmetry of each condition did not differ from zero (all *p* > 0.063, all dz < 0.496). Across all conditions, asymmetry at 0.5 Hz averaged −1.61 % ± 2.71 % (min: −12.25 %; max: 11.36 %). Overall, this shows that assessing asymmetry at 0.5 Hz was not influenced by the type of EVS stimulus or current intensity.

#### 3.3.4 Torsion at 2 Hz: Average-of-Sine vs pure 2 Hz sines

In the following analyses, torsion amplitude measured from the 2 Hz frequency components of the average-of-sine stimuli was compared to torsion evoked by “pure” sines at 2 Hz. As expected, torsion evoked from the 2 Hz component of the average-of-sines stimulus was smaller than that evoked by the pure 2 Hz sine. This is because each component in the average-of-sines had a reduced amplitude compared to the single-frequency stimulus.

The torsion data (Fig. 10D and E) revealed an EVS Stimulus X Current Intensity interaction (*p* < 0.001, $\eta_{p}^{2}$ = 0.618). This further revealed that, at 2 mA, torsion evoked with pure sines at 2 Hz (0.23 ± 0.01°) was 35 % greater than when measured from the 2 Hz component of average-of-sine stimuli (0.17 ± 0.01°; *p* < 0.001, dz = 1.380). Similarly, at 4 mA, torsion evoked with pure sines at 2 Hz (0.45 ± 0.01°) was 30 % greater than when measured from the 2 Hz component of average-of-sine stimuli (0.34 ± 0.01°; *p* = 0.005; dz = 1.912). These torsion values are consistent with those reported under Section 3.3.1 and reflect condition-specific means analysed separately for 2 mA and 4 mA due to a significant interaction. Overall, this shows that torsion evoked by pure sines at 2 Hz was 30 to 35 % greater than the 2 Hz components of average-of-sine stimuli, as expected due to the difference in stimulus magnitude.

#### 3.3.5 Asymmetry at 2 Hz was not influenced by EVS stimuli or current intensity

When measured from the bandpass-filtered 2 Hz frequency components, the asymmetry data (Fig. 10F) revealed no EVS Stimulus X Current Intensity interaction (*p* = 0.690, $\eta_{p}^{2}$ = 0.007), no effect of EVS Stimulus (*p* = 0.195, $\eta_{p}^{2}$ = 0.072), and no effect of Current Intensity (*p* = 0.892, $\eta_{p}^{2}$ = 0.001). Moreover, the mean asymmetry of each condition did not differ from zero (all *p* > 0.395, all dz < 0.308). Across all conditions, asymmetry at 2 Hz averaged −3.35 % ± 2.54 % (min: −17.24 %; max: 10.51 %). Overall, this shows that assessing asymmetry based on 2 Hz frequency components is not influenced by the type of EVS stimulus or current intensity.

#### 3.3.6 Sines at 0.5 Hz evoked the least discomfort

**Fig. 11:**
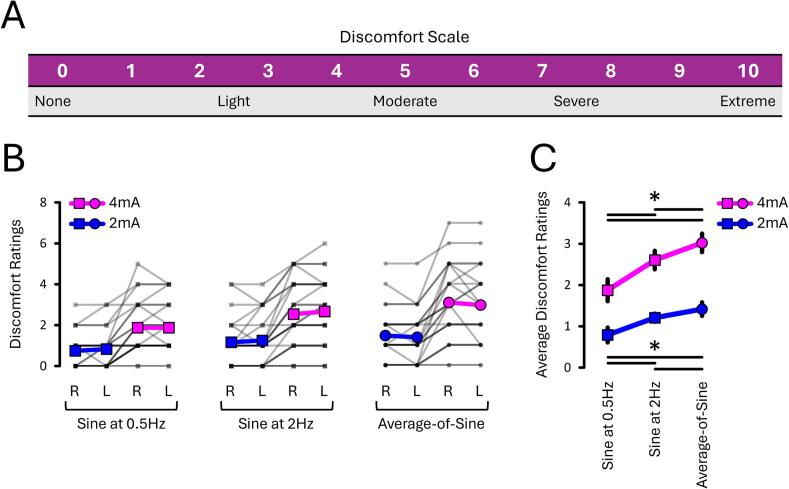
**Perceived discomfort ratings from EXP#3.** Panel **A** shows the visual analogue scale used by participants to rate discomfort after each trial. Panels **B–C** show discomfort ratings across stimulus frequency, current intensity, and stimulation side. Discomfort was lowest for sines at 0.5 Hz and increased with sines at 2 Hz and with average-of-sine stimuli, particularly at 4 mA. Grey lines show individual data; bold coloured lines indicate condition means. Asterisks (*) denote significant differences (p < 0.05). “R” and “L” = right- and left-sided stimulation, respectively.

The perceived discomfort data (Fig. 11B and C) selectively revealed an EVS Stimulus X Current Intensity interaction (*p* = 0.022, $\eta_{p}^{2}$ = 0.152). This further revealed that, for both 2 mA and 4 mA, discomfort was lower for sines at 0.5 Hz, compared to either 2 Hz (both *p* < 0.005, both dz > 0.667) or the average-of-sine stimuli (both *p* < 0.003, both dz > 0.758). For both 2 mA and 4 mA, discomfort at 2 Hz was also lower than with average-of-sine stimuli (both *p* < 0.041, both dz > 0.409). Overall, this shows that sines at 0.5 Hz generated the least discomfort, whilst the average-of-sine stimuli yielded the greatest discomfort.

#### 3.3.7 Assessing asymmetry with sines at 0.5 Hz required 300 % more time than with sines at 2 Hz

**Fig. 12:**
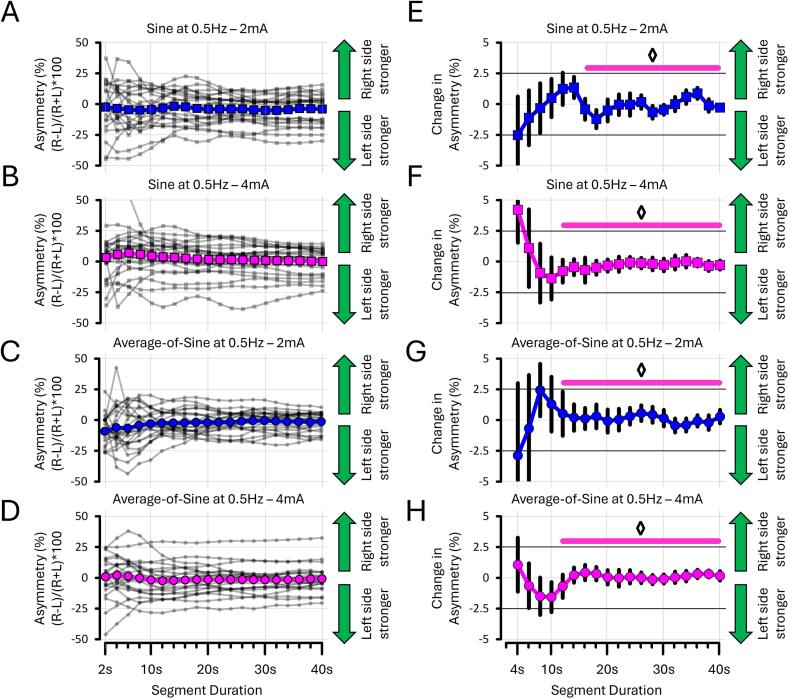
**Asymmetry at 0.5 Hz by segment duration from EXP#3.** Panels show asymmetry **(A–D)** and differentiated asymmetry **(E–H)** for sines at 0.5 Hz and the 0.5 Hz frequency components of average-of-sine stimuli. All asymmetry estimates stabilised within 20 s. Diamonds (◊) indicate when differentiated asymmetry stabilised within ± 2.5 % around zero. Grey lines show individual data; bold coloured lines indicate condition means.

**Fig. 13:**
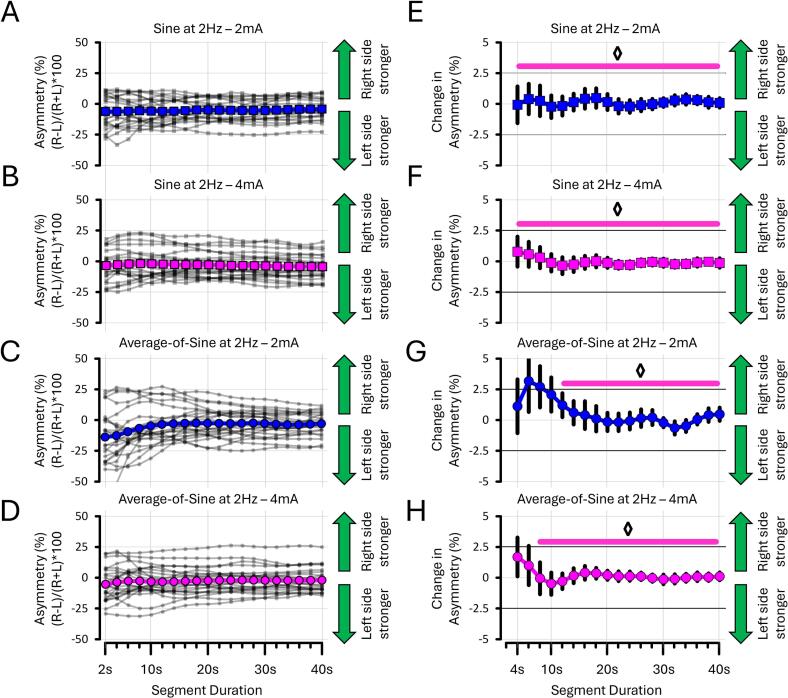
**Asymmetry at 2 Hz by segment duration from EXP#3.** Panels show asymmetry **(A–D)** and differentiated asymmetry **(E–H)** for sines at 2 Hz and the 2 Hz frequency components of average-of-sine stimuli. In contrast to 0.5 Hz, asymmetry stabilised more quickly with sines at 2 Hz. Nevertheless, all asymmetry measures stabilised within 20 s. Diamonds (◊) indicate when differentiated asymmetry stabilised within a change value of 0 ± 2.5 %. Grey lines show individual data; bold coloured lines indicate condition means. “R” and “L” = right- and left-sided stimulation, respectively.

The following analyses sought to determine the shortest stimulation duration (in seconds) necessary to obtain a stable asymmetry assessment. Torsion data were prepared as described in Section 2.4.3. The results presented here are based on descriptive statistics only (see Supplementary Table 1 for details). The data revealed that 12 to 16 s are required for asymmetry to stabilise when using sines at 0.5 Hz (Fig. 12A-B-E-F). In contrast, sines at 2 Hz required only 4 s to achieve a similar outcome (Fig. 13A-B-E-F). This suggests that low-frequency stimuli require approximately 300 % more time to reach stable asymmetry values than higher-frequency ones. For the average-of-sine stimuli, the 0.5 Hz and 2 Hz frequency components required at least 12 and 8 s, respectively, to yield stable asymmetry estimates (Figs. 12C-D-G-H and 13C-D-G-H). This suggests that average-of-sine stimuli do not further reduce the stimulation duration to obtain stable asymmetry assessments. Taken together, these findings indicate that asymmetry values stabilise after 20 s of stimulation, regardless of the EVS stimulus used.

### 3.4 Normative data from the results of all three experiments

**Fig. 14:**
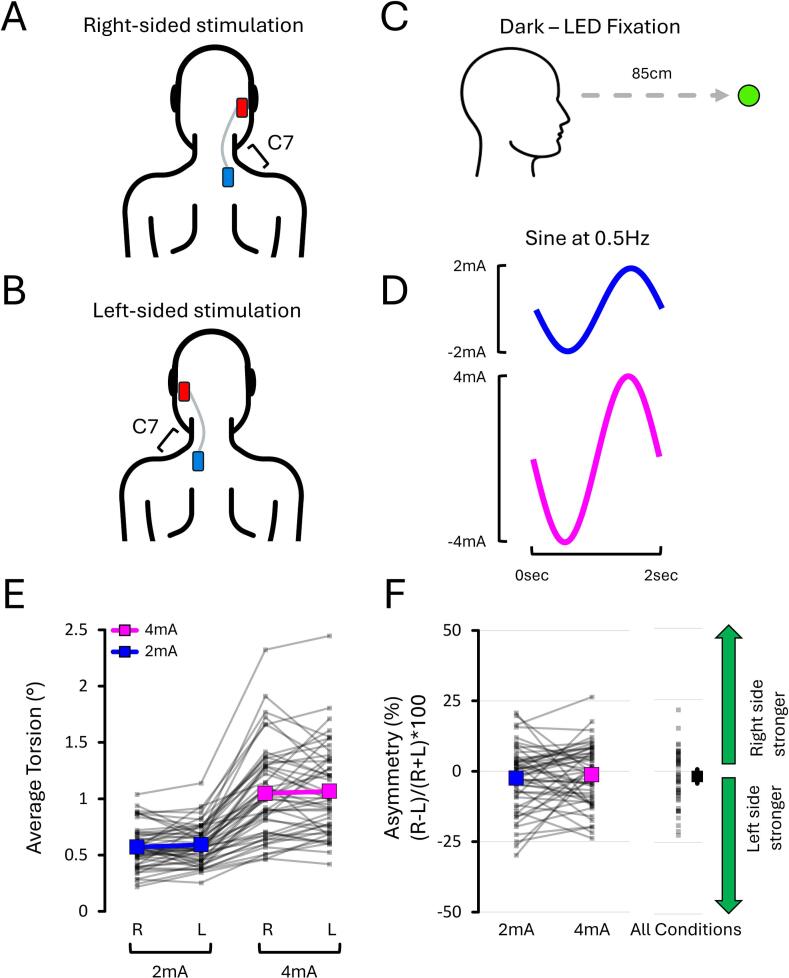
**Pooled torsion and asymmetry results (n = 53) from the condition common to all three experiments:** mastoid–C7 monoaural montage **(A–B)**, LED fixation in complete darkness **(C)**, and sines at 0.5 Hz delivered at 2 mA and 4 mA **(D)**. Torsion **(E)** averaged 0.58 ± 0.05° at 2 mA (min: 0.27°; max: 1.09°) and 1.06 ± 0.10° at 4 mA (min: 0.44°; max: 2.38°). Mean asymmetry **(F)** across both intensities averaged –1.87 % ± 2.48 % (min: –22.40 %; max: 21.46 %) and did not differ from zero. Grey lines show individual data; bold coloured lines indicate condition means. “R” and “L” = right- and left-sided stimulation, respectively.

One secondary objective of this work was to provide normative data on torsion and asymmetry. To do so, data from the condition common to all three experiments were pooled: mastoid-C7 monoaural montage (Fig. 14A and B), LED fixation in darkness (Fig. 14C), and sines at 0.5 Hz delivered at 2 mA and 4 mA (Fig. 14D). This resulted in 53 independent data sets, which were analysed using 2 Stimulation Side (Right, Left) X 2 Current Intensity (2 mA, 4 mA) rmANOVAs.

The torsion data (Fig. 14E) selectively revealed an effect of Current Intensity (*p* < 0.001, $\eta_{p}^{2}$ = 0.789). This revealed that torsion at 2 mA (0.58 ± 0.05°; min: 0.27°; max: 1.09°) was 55 % smaller than at 4 mA (1.06 ± 0.10°; min: 0.44°; max: 2.38°). Moreover, the asymmetry data (Fig. 14F) revealed no difference between asymmetry at 2 mA and 4 mA (*p* = 0.472, dz = -0.099). Lastly, the mean asymmetry of each condition did not differ from zero (both *p* > 0.241, both dz < 0.217). When averaged across both current intensities, asymmetry averaged −1.87 ± 2.48 % (min: –22.40 %; max: 21.46 %). Overall, these results provide normative values for EVS-evoked torsion and asymmetry and suggest that a cutoff of approximately ± 25 % could be used to identify pathological asymmetry.

## 4 Discussion

The objective of this work was to investigate how various stimulus parameters influence the amplitude of EVS-evoked ocular torsion responses and the assessment of interaural asymmetry. The results revealed the following. First, fixating on an LED with ambient lights decreased the size of torsion by at least 20 % compared to darkness, suggesting that darkness is preferable for maximising torsional response amplitude. Second, when the reference electrode was positioned next to C7 (i.e., using a mastoid-C7 montage), torsional responses to monoaural stimulation reached a floor effect at 50 % of the amplitude observed during binaural stimulation. This suggests that the mastoid-C7 montage effectively redirects current away from the contralateral (unstimulated) vestibular system. As such, this montage appears suitable for assessing vestibular function separately in each ear. Third, sinusoidal stimuli at a frequency of 0.5 Hz evoked torsion responses 250–275 % larger than 2 Hz stimuli but required 300 % longer duration (12 s vs 4 s) to achieve a stable asymmetry value. As such, sines at 0.5 Hz may be preferrable for assessing residual vestibular function, whereas sines at 2 Hz may be better suited for assessing asymmetry. Finally, pooled results (n = 53) revealed that asymmetry at 2 mA and 4 mA averaged 0 % and ranged between –22.4 % and 21.5 %. This suggests that a cutoff of approximately ± 25 % can be used to identify pathological asymmetry. Overall, these results provide guidelines for assessing vestibular function using EVS.

### 4.1 Ambient lights attenuate ocular torsion by 20 to 25 % compared to complete darkness

One novel result is that fixating on an LED in the presence of ambient lights attenuated torsion by 20–25 % compared to darkness (with or without LED), which aligns with previous animal work (Straumann et al., 1992). One implication is that darkness maximises the size of torsion, thereby optimising the assessment of residual vestibular function using EVS (Vailleau et al., 2011). Another novel result is that estimates of interaural asymmetry did not differ whether a fixation LED was provided or not, and whether fixation occurred in darkness or with ambient lights. This suggests that asymmetry assessments are robust to the presence of a fixation LED and ambient light conditions.

A secondary finding is that LED fixation suppressed horizontal and vertical eye responses, consistent with previous work (Hirvonen et al., 2012, Korda et al., 2021; although see Severac et al., 2003). One advantage of providing a fixation LED is that it ensures the irises remain visible to the cameras, thereby optimising torsional tracking (Jin et al., 2020, Ong and Haslwanter, 2010). However, suppressing horizontal and vertical eye responses may conceal clinically relevant information. For instance, MacDougall et al. (2005) reported that EVS-evoked changes in 3D eye responses – including horizontal and vertical components – reflected the underlying pathophysiology of vestibular dysfunction. This suggests that torsion is not the only clinically informative response component. Here, our Fourier spectra revealed clear frequency-specific components in torsional responses that matched the delivered EVS stimulus, whereas horizontal and vertical responses lacked such frequency locking (see Fig. 4 and Supplementary Figs. 2, 4, and 6). This pattern suggests that our current stimulation parameters favour torsion as the most reliable response for clinical diagnosis. One possibility is that alternating sine stimuli limit the amplitude of translational responses (Mackenzie and Reynolds, 2018), which may reduce their suitability as clinical biomarkers under the current stimulation parameters. Altogether, this evidence highlights the importance of carefully selecting fixation strategies when using EVS, as they can influence the visibility of diagnostically relevant eye responses other than torsion.

### 4.2 A mastoid-C7 montage to preferentially stimulate a single vestibular system

One interesting result is that torsional responses were 50 % smaller with monoaural montages compared to binaural stimulation (see Fig. 8). Although expected (MacDougall et al., 2003), this result is important as it suggests that all monoaural montages effectively redirect most of the EVS current away from the “unstimulated” vestibular system (Thomas et al., 2020, Truong et al., 2024). Moreover, manipulating the electrode montage did not alter asymmetry, suggesting that asymmetry is robust to changes in electrode montage. However, torsional responses increased slightly when the reference electrode was moved from C7 to the sub-occipital location. This suggests that placing the reference electrode closer than C7 to the unstimulated mastoid may allow current to spread to the contralateral vestibular system, potentially compromising asymmetry estimates. In contrast, moving the electrode further away from C7 had no additional effect on torsion, indicating that response amplitude reached a floor value of 50 % of the maximum binaural value. Although this floor effect suggests that current spread is negligible, some degree of contralateral activation cannot be ruled out. This is because the precise EVS current flow patterns in humans remain unknown, as they are influenced by several factors: the symmetry of the electrode montage (Truong et al., 2024), electrode size (Truong et al., 2023), individual variability in vestibular anatomy (Johnson Chacko et al., 2018), and the electrical conductivity of human head tissues (McCann et al., 2019). Nevertheless, these results suggest that placing the reference electrode at or beyond C7 effectively minimises unwanted current spread to the contralateral (unstimulated) vestibular system. Altogether, this evidence underscores the importance of considering the electrode montage when interpreting EVS-evoked torsional responses.

It should be noted that the present results differ from previous EVS work conducted in vestibular-deafferented patients. Specifically, Aw et al. (2013) reported that using a mastoid-C7 montage to deliver constant-current pulses at 5 mA to the deafferented ears evoked torsional eye responses that depended on current polarity. This dependence on polarity is important because it suggests that current flowing through the deafferented ear or C7 spread to the healthy ear, thereby evoking torsion. However, these findings are partially inconsistent with the present results and those of Thomas et al. (2020), which indicate minimal current spread with a mastoid-C7 montage. One possible explanation for this discrepancy is that Aw et al. (2013) used a higher peak current (5  mA) than was used here (2 mA or 4 mA), potentially increasing the likelihood of current spreading to the unstimulated vestibular system. Future studies should clarify whether current intensity interacts with the electrode montage to modulate the extent of current spread.

### 4.3 Sines at 0.5 Hz to maximise torsion, but sines at 2 Hz to quickly assess asymmetry

Another novel result is that sines at 0.5 Hz evoked torsion 250 to 275 % greater than those evoked at 2 Hz. However, a minimum of 4 s of 4 mA stimulation was required to achieve a stable asymmetry assessment at 2 Hz, whereas at least 12 s were required when using 0.5 Hz stimuli – representing a 300 % increase in stimulation duration. This trade-off suggests that sines at 0.5 Hz may be better suited for assessing residual vestibular function (Vailleau et al., 2011), whereas sines at 2 Hz may be better for assessing asymmetry (Mackenzie et al., 2019). Interestingly, although asymmetry may stabilise after as little as 4 s at 2 Hz, it consistently stabilised after 20 s across all EVS stimuli. This suggests that clinicians should ideally stimulate for at least 20 s to ensure reliable asymmetry values. Moreover, in clinical settings, the choice of stimulation frequency may also depend on individual discomfort. Here, discomfort ratings were systematically greater at 4 mA than at 2 mA, and higher for 2 Hz than for 0.5 Hz stimuli (Fig. 11). Hence, reducing either current intensity or stimulus frequency improved comfort. Nevertheless, it is worth noting that discomfort was generally low for all stimuli, with ratings ≤ 3 on our 10-point scale. Altogether, these findings highlight the importance of tailoring EVS parameters to the clinical objective – whether maximising torsional amplitude, optimising asymmetry assessment, or minimising patient discomfort.

Combining sines at 0.5 Hz and 2 Hz into an average-of-sine stimulus decreased the size of torsion by 20–35 % for each bandpass-filtered frequency components. This waveform was constructed by averaging full-amplitude 0.5 Hz and 2 Hz sine waves without phase offset and then normalised to match the net energy (i.e., RMS current value) of the individual sine stimuli. This normalisation step was intended to comparably engage vestibular afferents while avoiding excessive peak currents. However, this energy-matching procedure reduced the effective current by ∼29 % at each frequency component (0.5 Hz and 2 Hz), consistent with the 20–35 % reductions in torsion after bandpass filtering. Such a current reduction would be expected to proportionately decrease torsional responses, given the largely linear relationship between EVS amplitude and VOR gain (Kwan et al., 2019). Nevertheless, average-of-sine stimuli did not allow a stable asymmetry measure to be reached more quickly than single-frequency stimuli delivered independently, suggesting they do not accelerate asymmetry assessments. Additionally, average-of-sine stimuli evoked greater discomfort than either pure sines at 0.5 Hz or 2 Hz, presumably because the peak current was ∼ 36 % greater (2.73 mA and 5.46 mA compared to 2 mA and 4 mA, respectively). Overall, these results suggest that combining two sine stimuli into a single waveform does not enhance response amplitude, reduce asymmetry assessment time, or reduce discomfort. However, in time-constrained settings where both 0.5 Hz and 2 Hz responses are required, an average-of-sine stimulus may still offer practical benefits by reducing the number of trials. In clinical contexts, delivering average-of-sine stimuli may therefore remain a viable option for vestibular assessment.

### 4.4 A ± 25 % cutoff could be used to identify pathological asymmetry using EVS

One novel result comes from pooling data (n = 53) from the experimental condition that was repeated across the three experiments: mastoid-C7 montage, LED fixation in darkness, sines at 0.5 Hz, and current intensities of 2 mA and 4 mA (Fig. 14). Torsional responses averaged 0.58 ± 0.05° (min: 0.27°; max: 1.09°) at 2 mA, and 1.06 ± 0.10° (min: 0.44°; max: 2.38°) at 4 mA. Note that these values represent the mean ± 95 % confidence intervals. Because this condition was repeated across experiments and included a large sample, it provides a valuable reference for expected torsional amplitude under clinically relevant conditions. Interestingly, previous work showed that tri-axial eye responses to EVS are reliable within individuals (MacDougall et al., 2002). This intra-individual reliability supports the potential of EVS for monitoring the return of patients toward normative values during vestibular rehabilitation therapy (Deveze et al., 2014, Han, 2021). In addition, pooled asymmetry estimates across 2 mA and 4 mA averaged −1.87 ± 2.48 % (min: –22.40 %; max: 21.46 %). The clustering of asymmetry values near 0 % was expected, as these data were drawn from young healthy adults. Nevertheless, these findings support the use of a ± 25 % cutoff to identify pathological asymmetry using EVS (Mackenzie et al., 2019), consistent with thresholds used in caloric irrigation testing (Shepard and Jacobson, 2016).

### 4.5 Limitations

One limitation is that EVS stimulus delivery was not synchronised with video-oculography recording, which was conducted using newly developed commercial equipment (Videonystagmography goggles; Interacoustics ©) and software (Ocular Counter-Roll test, MicroMedical VisualEyes; Interacoustics ©). This precluded measurement of latency or phase lag between the EVS stimuli and ocular response, which could offer additional insight into vestibular function. In vestibular patients, such metrics may help determine whether attenuated or absent responses reflect impaired encoding, transmission, or central integration of vestibular signals. Another limitation is that torsion amplitude was calculated as the average peak-to-peak amplitude measured from each cycle of the bandpass-filtered data. As such, a greater number of cycles was available when delivering EVS stimuli at 2 Hz (80 cycles) compared to 0.5 Hz (20 cycles) over 40 s. This could explain why a stable asymmetry measure was obtained in 4 s at 2 Hz (8 cycles), whereas 12 to 16 s were required at 0.5 Hz (6 to 8 cycles). One possibility is that at least eight cycles of ocular torsion must be evoked to reliably calculate asymmetry. In future studies, the number of cycles required to yield a stable asymmetry measurement should be examined more systematically. A further limitation is that this work was conducted on high signal-to-noise data from young healthy adults. Although appropriate in this context, measuring torsion on a cycle-by-cycle basis may be more susceptible to noise, particularly in patients with vestibular hypofunction (although see Mackenzie et al., 2019, Mackenzie et al., 2018). Whether alternative methods – such as fitting sine waves to the raw data or extracting torsion amplitude using fast Fourier transforms – could yield equivalent or more robust measurements under noisy conditions remains an open question. Finally, the extent to which the present results generalise to healthy older adults or individuals with vestibular disorders remains unclear and should be addressed in future studies.

## 5 Conclusion

The present results lead to the following recommendations for administering EVS. First, EVS should be delivered whilst participants fixate on an LED in darkness. This ensures stable iris tracking and optimises torsion amplitude. Second, a mastoid-C7 or mastoid-acromioclavicular montage should be used to limit current spread to the “unstimulated” vestibular system. This would ensure that EVS activates a single vestibular end organ. Third, to assess residual vestibular function, slower-frequency stimuli (i.e., sines at 0.5 Hz) are preferable as they evoke larger torsional responses. Fourth, to assess interaural asymmetry, higher-frequency stimuli (i.e., sines at 2 Hz) are recommended, as they yield stable asymmetry values more rapidly. Fifth, although shorter recordings may suffice when using higher-frequency stimuli, EVS-evoked eye responses should ideally be recorded for at least 20 s to ensure reliable measurement of torsion and asymmetry. Finally, clinicians should be aware of the important inter-individual variability in EVS-evoked responses, and that a cutoff of ± 25 % may be appropriate for identifying pathological asymmetry.

## Author contributions

R.H. and R.F.R. designed the project. R.H., S.P., and P.G., collected the data. R.H. analysed the data, prepared the figure, and wrote and revised the manuscript. R.H., R.I., D.W., and R.F.R. revised the manuscript.

## Declaration of Competing Interest

The authors declare that they have no known competing financial interests or personal relationships that could have appeared to influence the work reported in this paper.
