# Supplementary material for: Optimising electrical vestibular stimulation (EVS) for assessing vestibular function
Source: Clin Neurophysiol Pract. 2025 Sep 5;10:359–77. doi: 10.1016/j.cnp.2025.08.006 (PMC12747185; doi:10.1016/j.cnp.2025.08.006)
Supplement: Supplementary Data 1 [file mmc1.docx]

**Supplementary Results**

**EXP#1 – EFFECTS OF LIGHT**

The supplementary figures below show the average reconstructed torsional (Supplementary Figure 1), horizontal (Supplementary Figure 3), and vertical eye responses (Supplementary Figure 5) for each condition of EXP#1. The Fourier spectra of the full 40-second segments of reconstructed torsional (Supplementary Figure 2), horizontal (Supplementary Figure 4), and vertical eye responses (Supplementary Figure 6) are also shown below.


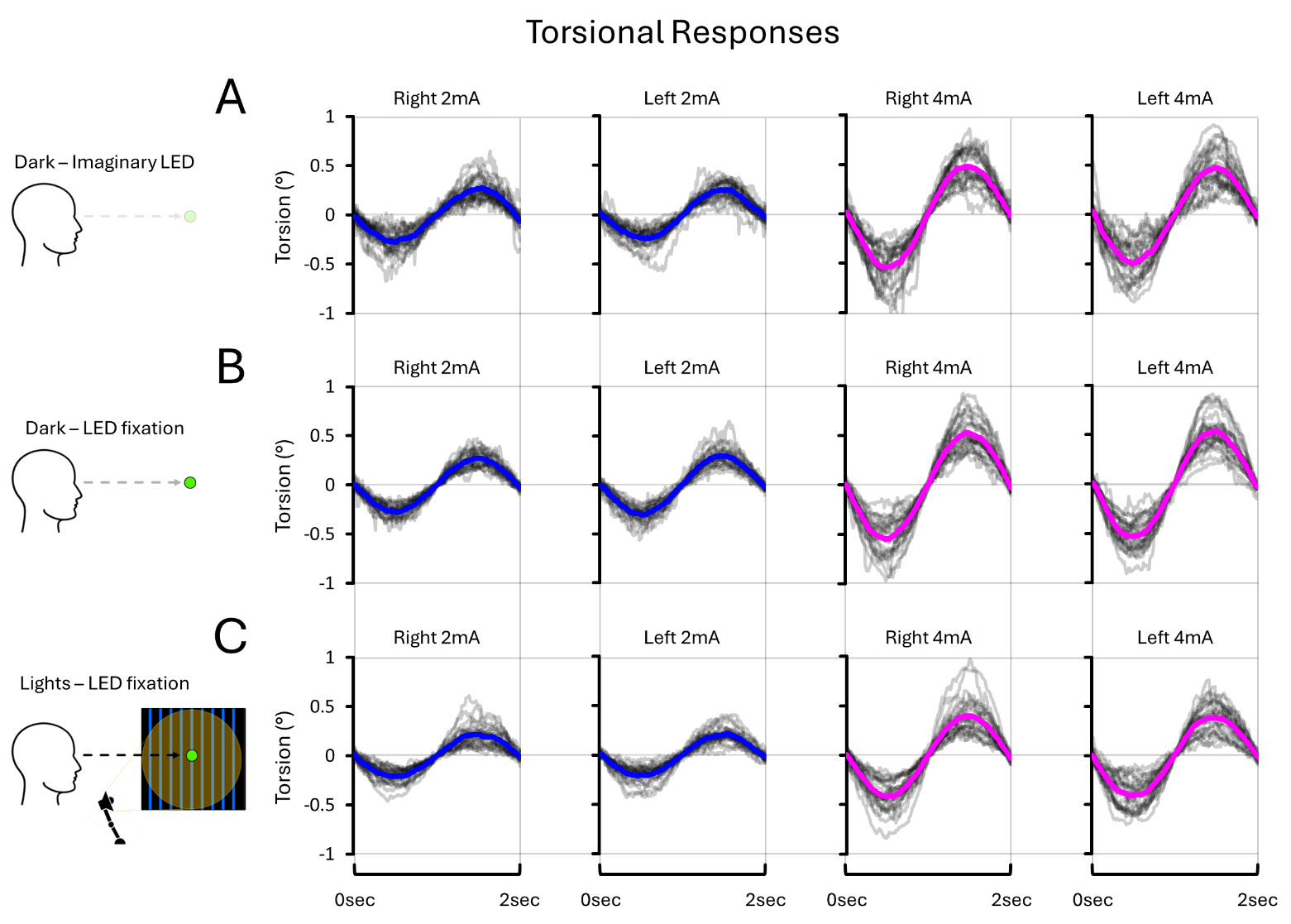


**Supplementary Figure 1. Torsional responses from EXP#1 across three light conditions.** Panels show responses during fixation on an imaginary LED in darkness **(A)**, an illuminating LED in darkness **(B)**, and an illuminating LED with ambient lights **(C)**, during 2mA and 4mA with a mastoid–C7 monoaural montage. Grey lines show individual data; the blue or magenta line indicates the condition mean. Positive values reflect clockwise torsion from the participant’s perspective.


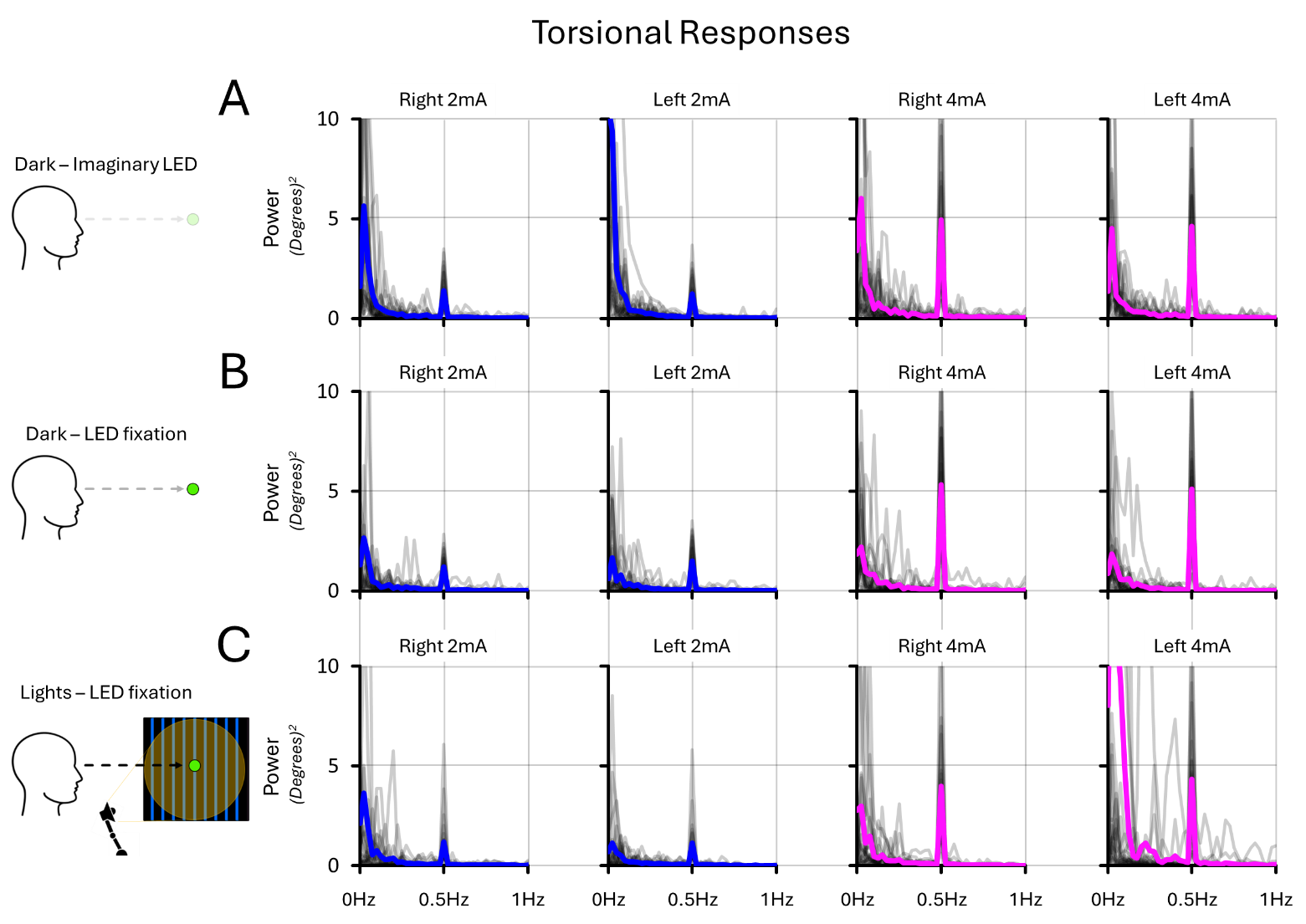


**Supplementary Figure 2. Fourier spectra of torsional responses from EXP#1 across three light conditions.** Panels show responses during fixation on an imaginary LED in darkness **(A),** an illuminating LED in darkness **(B),** and an illuminating LED with ambient lights **(C)**, during 2mA and 4mA with a mastoid–C7 monoaural montage. Grey lines show individual data; the blue or magenta line indicates the condition mean.


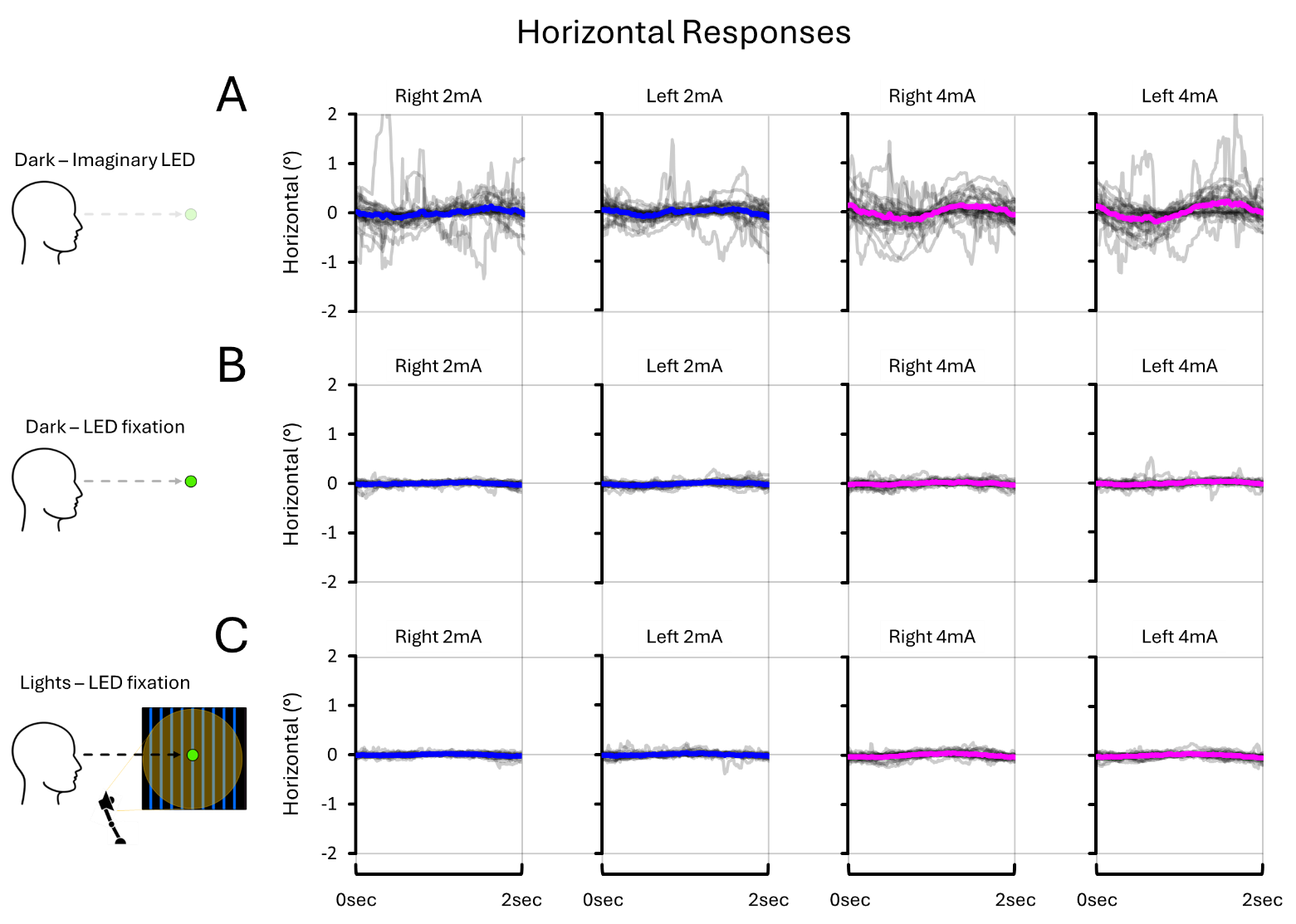


**Supplementary Figure 3. Horizontal responses from EXP#1 across three light conditions.** Panels show responses during fixation on an imaginary LED in darkness **(A)**, an illuminating LED in darkness **(B)**, and an illuminating LED with ambient lights **(C)**, during 2mA and 4mA with a mastoid–C7 monoaural montage. Grey lines show individual data; the blue or magenta line indicates the condition mean. Positive values reflect rightward motion from the participant’s perspective.


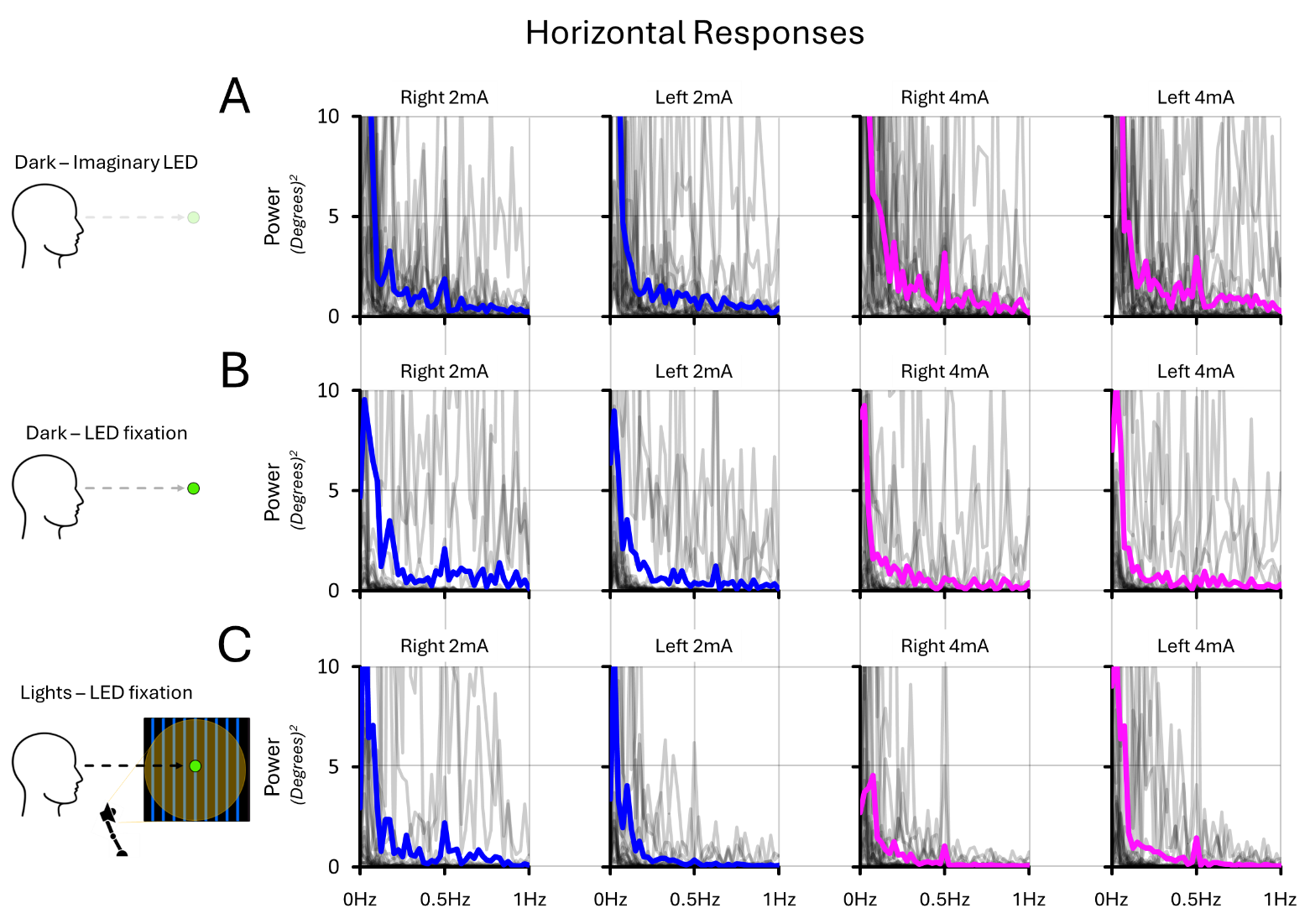


**Supplementary Figure 4. Fourier spectra of horizontal responses from EXP#1 across three light conditions.** Panels show responses during fixation on an imaginary LED in darkness **(A)**, an illuminating LED in darkness **(B)**, and an illuminating LED with ambient lights **(C)**, during 2mA and 4mA with a mastoid–C7 monoaural montage. Grey lines show individual data; the blue or magenta line indicates the condition mean.


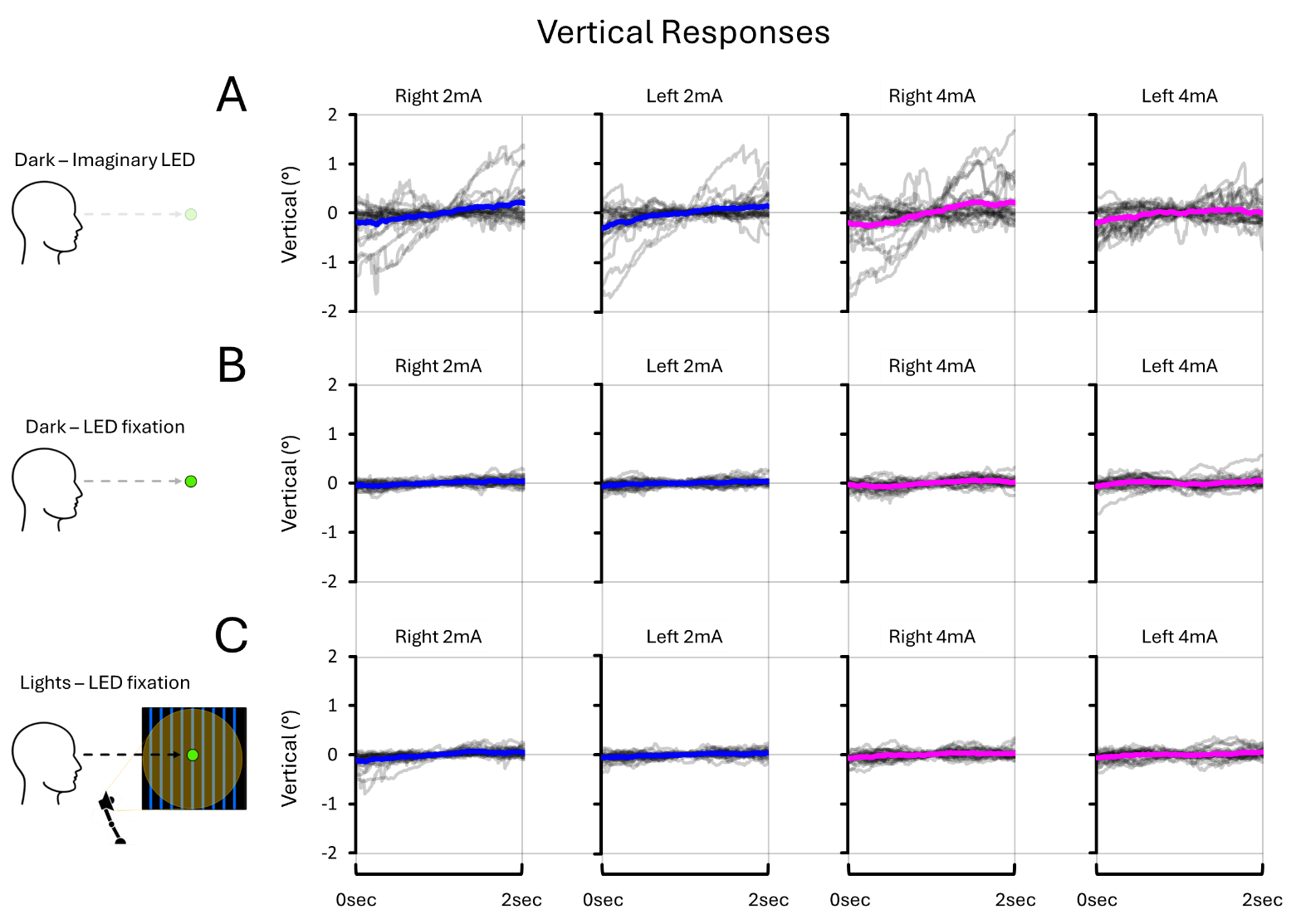


**Supplementary Figure 5. Vertical responses from EXP#1 across three light conditions**. Panels show responses during fixation on an imaginary LED in darkness **(A)**, an illuminating LED in darkness **(B)**, and an illuminating LED with ambient lights **(C)**, during 2mA and 4mA with a mastoid–C7 monoaural montage. Grey lines show individual data; the blue or magenta line indicates the condition mean. Positive values reflect upward motion from the participant’s perspective. Vertical responses from the right eye were inverted to align with left-eye conventions (see Methods).


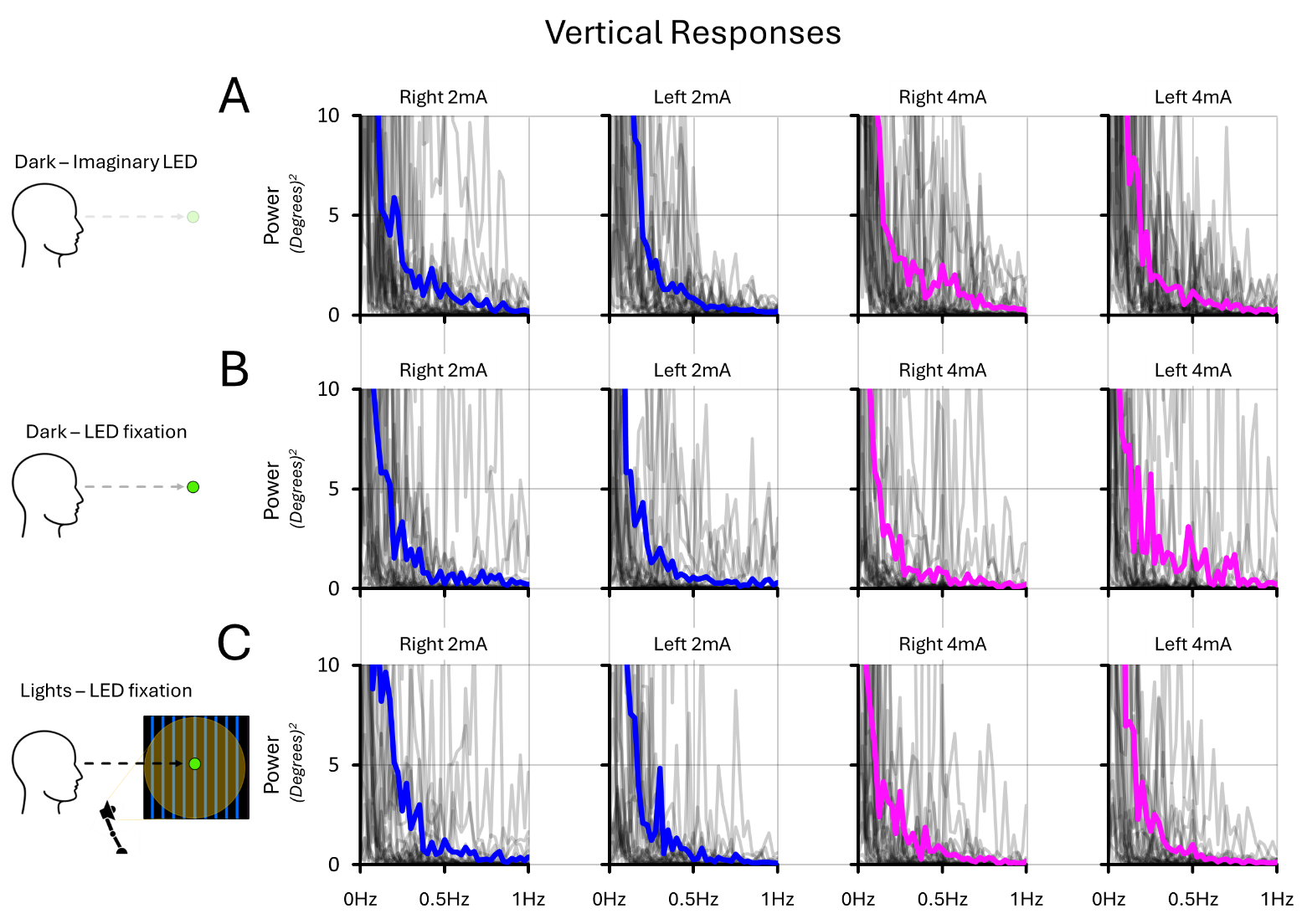


**Supplementary Figure 6. Fourier spectra of vertical responses from EXP#1 across three light conditions.** Panels show responses during fixation on an imaginary LED in darkness **(A)**, an illuminating LED in darkness **(B)**, and an illuminating LED with ambient lights **(C)**, during 2mA and 4mA with a mastoid–C7 monoaural montage. Grey lines show individual data; the blue or magenta line indicates the condition mean.

**EXP#2 – EFFECTS OF ELECTRODE MONTAGE**

The supplementary figures below show the average reconstructed torsional responses (Supplementary Figure 7) for each condition of EXP#2. The Fourier spectra of the full 40-second segments of torsional responses (Supplementary Figure 8) are also shown below.


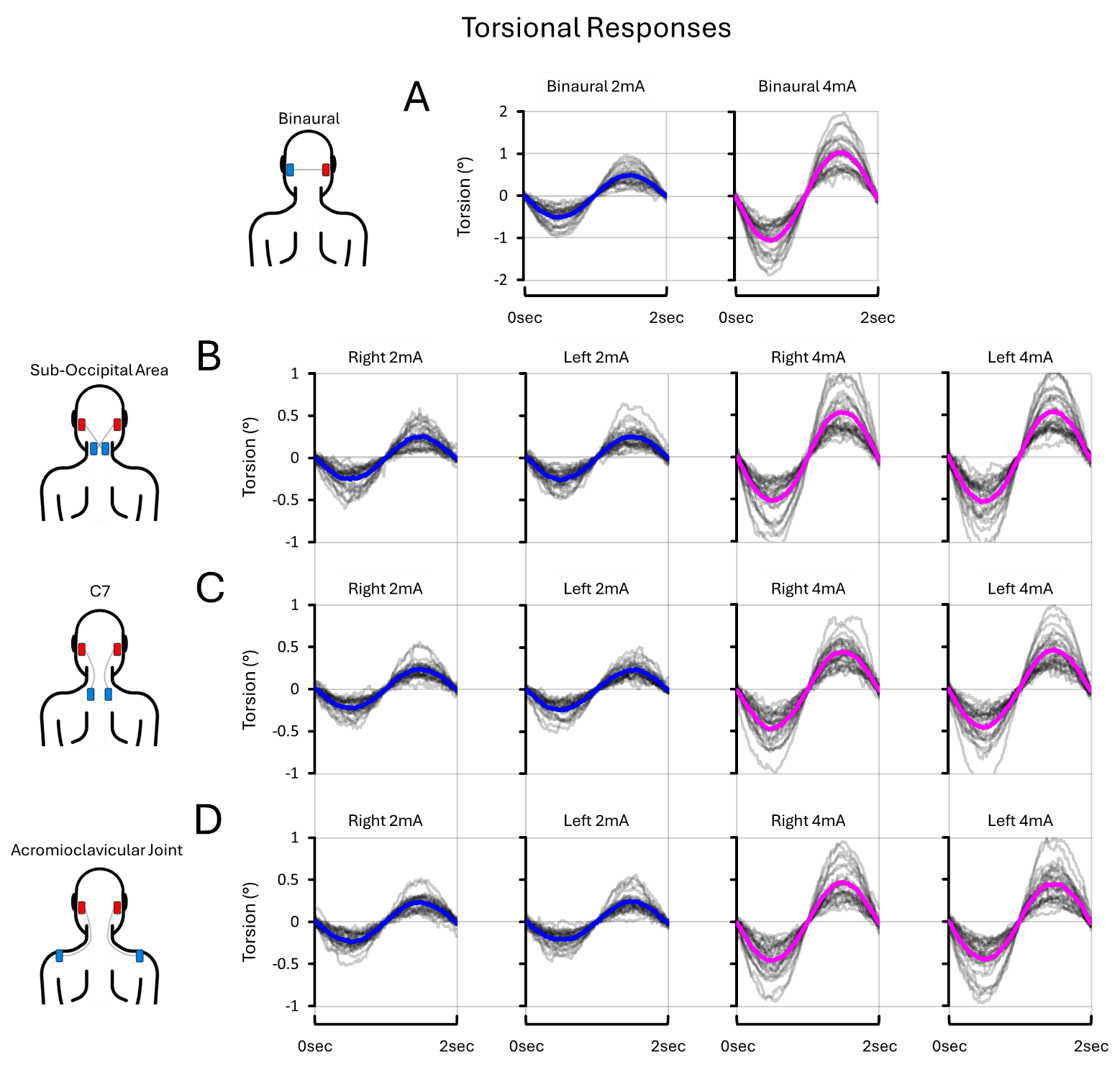


**Supplementary Figure 7. Torsional responses from EXP#2 across four electrode configurations.** Panels show responses during binaural **(A)**, mastoid–suboccipital **(B)**, mastoid–C7 **(C)**, and mastoid–acromioclavicular joint montage **(D)** stimulation, during 2mA and 4mA with LED fixation in darkness. Grey lines show individual data; the blue or magenta line indicates the condition mean. Positive values reflect clockwise torsion from the participant’s perspective.


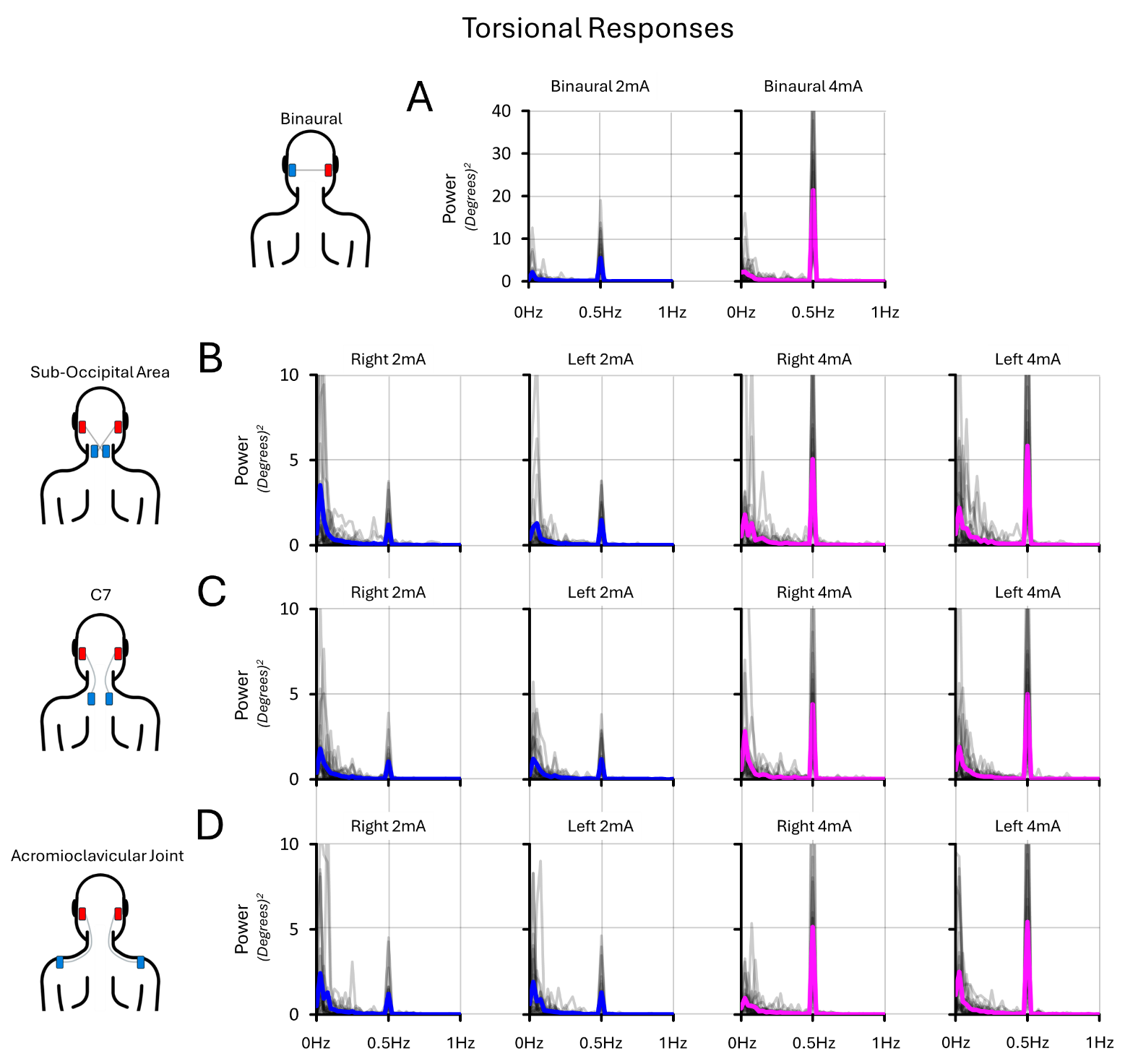


**Supplementary Figure 8. Fourier spectra of torsional responses from EXP#2 across four electrode montages.** Panels show responses during binaural **(A)**, mastoid–suboccipital **(B)**, mastoid–C7 **(C)**, and mastoid–acromioclavicular joint **(D)** stimulation, during 2mA and 4mA with LED fixation in darkness. Grey lines show individual data; the blue or magenta line indicates the condition mean.

**EXP#3 – EFFECTS OF SINUSOIDAL EVS STIMULI FREQUENCY**

The supplementary figures below show the average reconstructed torsional responses (Supplementary Figure 9) for each condition of EXP#3. The Fourier spectra of the full 40-second segments of torsional responses (Supplementary Figure 10) are also shown below.


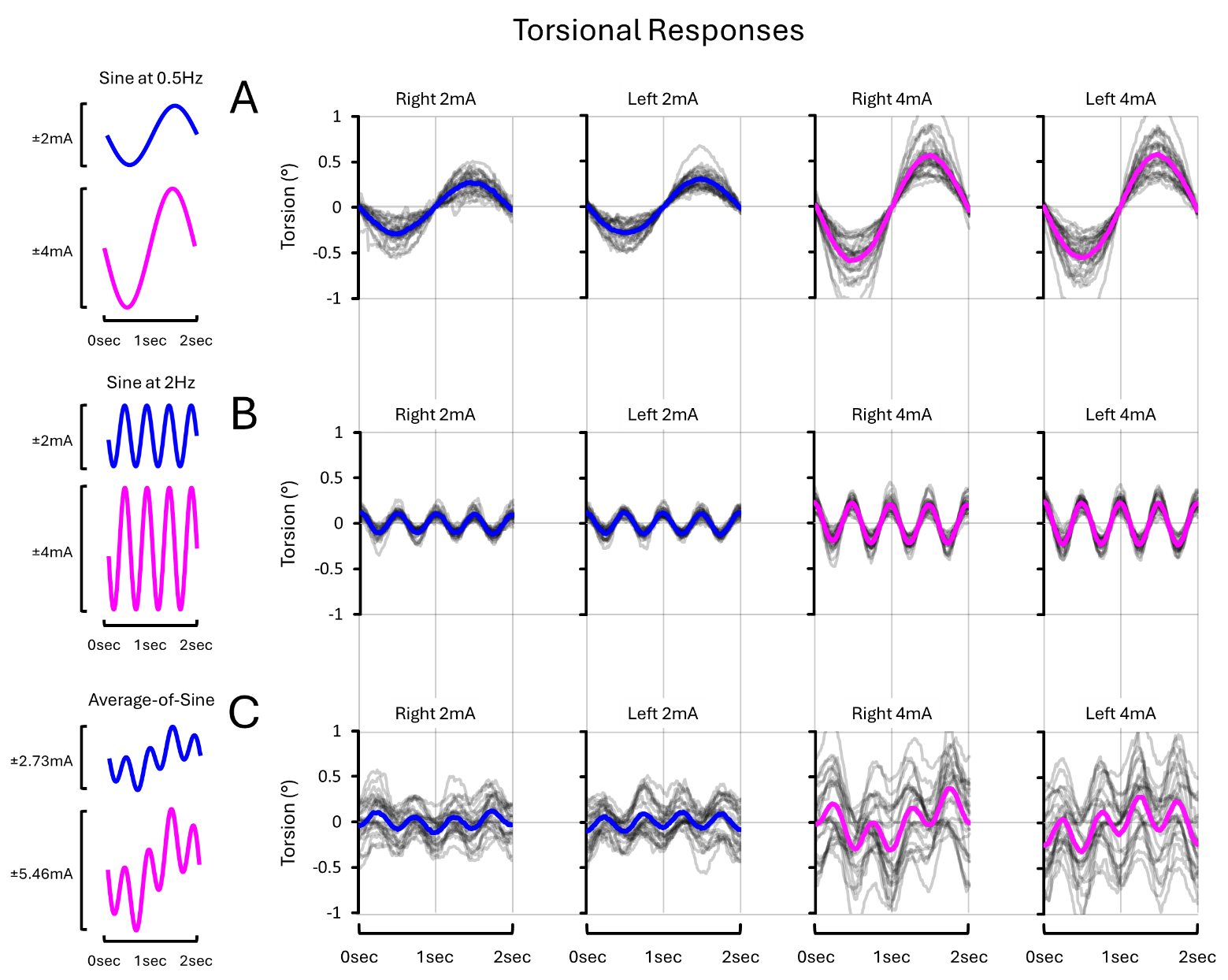


**Supplementary Figure 9. Torsional responses from EXP#3 across three EVS stimuli.** Panels show responses to sine at 0.5 Hz **(A)**, 2 Hz **(B)**, and average-of-sine stimuli **(C)**, during 2mA and 4mA using a mastoid–C7 monoaural montage. All conditions involved LED fixation in darkness. Grey lines show individual data; the blue or magenta line indicates the condition mean. Positive values reflect clockwise torsion from the participant’s perspective.


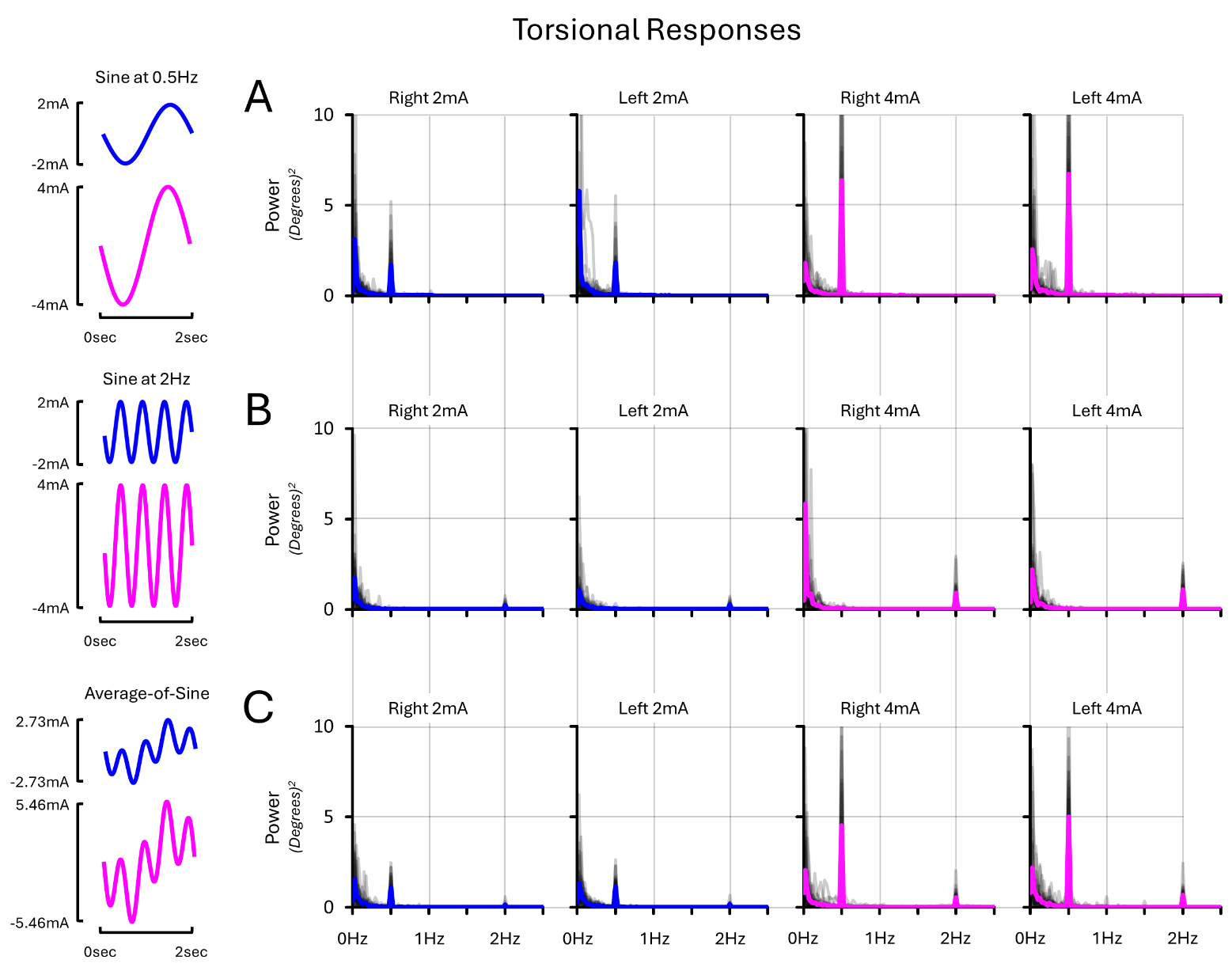


**Supplementary Figure 10. Fourier spectra of torsional responses from EXP#3 across three EVS stimuli.** Panels show responses to sine at 0.5 Hz **(A)**, 2 Hz **(B)**, and average-of-sine stimuli **(C)**, during 2mA and 4mA stimulation with a mastoid–C7 monoaural montage. All conditions involved LED fixation in darkness. Grey lines show individual data; the blue or magenta line indicates the condition mean.

**Supplementary Table 1 – Differentiated Asymmetry values by Segment Duration**

|  | **Sine at 0.5Hz** | | **Average-of-Sine at 0.5Hz** | | **Sine at 2Hz** | | **Average-of-Sine at 2Hz** | |
| --- | --- | --- | --- | --- | --- | --- | --- | --- |
|  | **2mA** | **4mA** | **2mA** | **4mA** | **2mA** | **4mA** | **2mA** | **4mA** |
| **4sec** | -2.53 ± 3.93 | 4.21 ± 2.67 | -2.88 ± 5.88 | 1.06 ± 2.19 | **-0.08 ± 1.50** | **0.78 ± 1.22** | 1.13 ± 2.18 | 1.69 ± 1.58 |
| **6sec** | -1.11 ± 3.15 | 1.09 ± 3.15 | -0.68 ± 4.34 | -0.64 ± 1.81 | **0.40 ± 1.23** | **0.56 ± 1.02** | 3.16 ± 2.50 | 1.00 ± 1.59 |
| **8sec** | -0.32 ± 2.22 | -0.95 ± 2.39 | 2.44 ± 2.13 | -1.50 ± 1.52 | **0.24 ± 1.24** | **0.30 ± 0.86** | 2.70 ± 1.68 | **-0.04 ± 1.30** |
| **10sec** | 0.50 ± 2.02 | -1.38 ± 1.71 | 1.30 ± 2.22 | -1.57 ± 1.22 | **-0.23 ± 0.92** | **-0.12 ± 0.70** | 2.08 ± 1.39 | **-0.48 ± 0.90** |
| **12sec** | 1.24 ± 1.55 | **-0.78 ± 0.93** | **0.52 ± 1.79** | **-0.69 ± 0.94** | **-0.18 ± 0.77** | **-0.35 ± 0.68** | **1.21 ± 0.97** | **-0.28 ± 0.63** |
| **14sec** | 1.35 ± 1.31 | **-0.46 ± 0.65** | **0.21 ± 1.07** | **0.22 ± 0.62** | **0.14 ± 0.68** | **-0.27 ± 0.60** | **0.60 ± 0.96** | **0.17 ± 0.61** |
| **16sec** | **-0.42 ± 0.86** | **-0.70 ± 1.01** | **0.12 ± 0.86** | **0.43 ± 0.62** | **0.42 ± 0.80** | **-0.09 ± 0.53** | **0.41 ± 1.13** | **0.40 ± 0.55** |
| **18sec** | **-1.22 ± 0.85** | **-0.50 ± 0.57** | **0.30 ± 0.97** | **0.33 ± 0.54** | **0.48 ± 0.79** | **0.00 ± 0.50** | **0.11 ± 1.03** | **0.37 ± 0.44** |
| **20sec** | **-0.52 ± 0.74** | **-0.33 ± 0.5** | **-0.08 ± 0.94** | **0.04 ± 0.59** | **0.16 ± 0.65** | **-0.12 ± 0.41** | **-0.15 ± 0.74** | **0.20 ± 0.38** |
| **22sec** | **-0.05 ± 0.89** | **-0.22 ± 0.61** | **0.04 ± 0.88** | **-0.03 ± 0.68** | **-0.20 ± 0.58** | **-0.29 ± 0.32** | **-0.18 ± 0.71** | **0.12 ± 0.39** |
| **24sec** | **-0.04 ± 0.84** | **-0.07 ± 0.45** | **0.31 ± 0.68** | **0.08 ± 0.54** | **-0.21 ± 0.51** | **-0.29 ± 0.29** | **-0.08 ± 0.69** | **0.13 ± 0.37** |
| **26sec** | **0.17 ± 0.91** | **-0.14 ± 0.42** | **0.56 ± 0.63** | **0.00 ± 0.44** | **-0.10 ± 0.50** | **-0.13 ± 0.28** | **0.16 ± 0.72** | **0.11 ± 0.30** |
| **28sec** | **-0.66 ± 0.55** | **-0.19 ± 0.51** | **0.43 ± 0.65** | **-0.14 ± 0.37** | **-0.02 ± 0.42** | **-0.06 ± 0.28** | **0.21 ± 0.61** | **-0.03 ± 0.30** |
| **30sec** | **-0.46 ± 0.52** | **-0.28 ± 0.51** | **0.15 ± 0.67** | **-0.10 ± 0.37** | **0.11 ± 0.35** | **-0.11 ± 0.33** | **-0.21 ± 0.38** | **-0.13 ± 0.39** |
| **32sec** | **0.00 ± 0.41** | **-0.08 ± 0.57** | **-0.44 ± 0.56** | **0.04 ± 0.46** | **0.28 ± 0.42** | **-0.23 ± 0.29** | **-0.63 ± 0.54** | **-0.10 ± 0.37** |
| **34sec** | **0.57 ± 0.58** | **0.02 ± 0.50** | **-0.40 ± 0.53** | **0.14 ± 0.43** | **0.36 ± 0.43** | **-0.20 ± 0.28** | **-0.48 ± 0.49** | **0.00 ± 0.32** |
| **36sec** | **0.87 ± 0.56** | **-0.09 ± 0.31** | **-0.07 ± 0.46** | **0.31 ± 0.34** | **0.31 ± 0.34** | **-0.10 ± 0.34** | **0.06 ± 0.41** | **0.08 ± 0.36** |
| **38sec** | **-0.11 ± 0.45** | **-0.36 ± 0.40** | **-0.20 ± 0.60** | **0.32 ± 0.28** | **0.13 ± 0.44** | **-0.04 ± 0.22** | **0.40 ± 0.55** | **0.04 ± 0.31** |
| **40sec** | **-0.28 ± 0.51** | **-0.28 ± 0.37** | **0.28 ± 0.55** | **0.15 ± 0.38** | **0.08 ± 0.38** | **-0.14 ± 0.38** | **0.48 ± 0.54** | **0.11 ± 0.32** |

For all cells in the table, the unit of measure is a percentage (%). The descriptive statistics represent the mean ± 95% CIs. The green-coloured cells with the bold font indicate the segment duration for which the differentiated asymmetry stabilised at a change value of 0% ± 2.5%.
